# Low, plasma level‑informed native curcumin concentrations fail to induce cell death in human lung and colorectal cancer cells

**DOI:** 10.1080/13880209.2026.2640678

**Published:** 2026-03-19

**Authors:** Ilma Imtiaz, Janet Schloss, Andrea Bugarcic

**Affiliations:** National Centre for Naturopathic Medicine, Southern Cross University, Lismore, Australia

**Keywords:** Curcumin, caspase 3, apoptosis, Cyclin D1, lung cancer, colorectal cancer

## Abstract

**Background/objectives:**

Curcumin, a dietary polyphenol derived from turmeric, has been widely studied for its anti-cancer properties, yet its effects at clinically relevant concentrations remain unclear. This study investigates the anti-cancer effects of curcumin at low *in vitro* concentrations selected based on reported plasma ranges using *in vitro* lung and CRC models, with a focus on underlying cellular mechanisms.

**Methods:**

Curcumin was tested at 4, 10, 20, and 50 µg/mL in two CRC cell lines (Caco-2 and HT29) and two lung cancer cell lines (A549 and H460).

**Results:**

MTS assays showed that at a low concentration of Curcumin 4 µg/mL, cell viability remained above 100% across all cell lines (A549: 102.1%, H460: 101.1%, Caco-2: 103.6%, HT29: 104.9%, *n* = 3, *p* > 0.05) and had no significant effect on cell death. Immunofluorescence analysis showed increased nuclear Cyclin D1 levels at 4 µg/mL curcumin in H460 and HT29 cells (*p* < 0.001), and no change in Caco-2 cells (*p* = 0.17), and a significant reduction in A549 cells (*p* < 0.001), suggesting promotion of cell cycle progression in H460 and HT29 cells only. Western blotting analysis showed higher levels of procaspase-3 without evidence of cleavage at 4 µg/mL, indicating the absence of apoptosis. A reduction in procaspase 3 levels were observed at 20 µg/mL (Caco-2, *p* < 0.05) and 50 µg/mL (H460, *p* < 0.05; A549, and HT29, *p* > 0.05).

**Conclusions:**

These findings suggest that at low. plasma level-informed concentrations, curcumin may support cancer cell survival rather than induce cytotoxicity. This study highlights the need for further pre-clinical evaluation of polyphenols at clinically relevant concentrations.

## Introduction

Cancer remains a leading cause of mortality worldwide and a major global health burden (Sung et al. 2021; Bray et al. 2024). Among all types, lung and colorectal cancer (CRC) account for the highest number of deaths in 2022, with approximately 1.8 million and 900,000 annual fatalities, respectively (Bray et al. 2024), and in 2024 lung and CRC remained the top causes of cancer deaths nationally in Australia (Cancer Australia, 2025; Australian Institute of Health and Welfare, 2019). These cancers are driven by genetic and epigenetic alterations that disrupt signaling pathways, leading to tumor initiation, progression, and metastasis—the primary cause of cancer-related death. For example, in Non-Small Cell Lung Cancer (NSCLC), mutation alterations that have been found in driver genes include *EGFR*, *ALK*, *ROS1*, *KRAS*, *BRAF*, *MET*, and *HER2* (Grodzka et al. 2023). Similarly, in approximately 90% of CRC cases, dysregulation of the MAPK/PI3K, Wnt/β-catenin, TP53, and TGF-β pathways has been found (Arvelo et al. 2015; Fois et al. 2021). These signaling disruptions can promote uncontrolled proliferation, invasion, and resistance to apoptosis (Mármol et al. 2017; Kang et al. 2021).

Despite advances in surgery, chemotherapy, and radiotherapy, current treatments are often associated with significant side effects and variable outcomes (Zhou et al. 2013). As a result, there is growing interest in complementary approaches that may reduce treatment-related toxicity or enhance therapeutic outcomes, including the use of herbal medicines. Phytochemicals have shown promise in sensitizing tumors to conventional therapies, reducing treatment-related toxicity, and improving patient quality of life (Monteiro et al. 2014). Examples of these include crocetin, an active compound derived from the *Crocus sativus L.* (saffron) flower, has been shown to induce apoptosis through different pathways in CRC cell lines by activating the intrinsic (mitochondrial) pathway in HCT116 cells and the extrinsic (death receptor) pathway in HT29 cells (Ray et al. 2016). Similarly, piperine, an amide extracted from *Piper nigrum* (black pepper), has demonstrated cytotoxicity at concentrations above 100 µM in A549 lung cancer cells and disruption of epithelial–mesenchymal transition (EMT)-related processes by inhibiting ERK1/2 and SMAD2 phosphorylation (Marques da Fonseca et al. 2020).

*Curcuma longa* L. (turmeric), a widely used traditional medicinal herb, contains curcumin, a yellow phenolic compound with well-documented anti-inflammatory and anti-cancer properties (Rashad 2025). Curcumin has demonstrated anti-cancer effects across various cancers, including lung and colorectal, by modulating key pathways such as MAPK, NF-κB, PI3K/Akt, and Wnt/β-catenin (Bhatia et al. 2021; Imtiaz et al. 2025). Pharmacokinetic studies have shown that oral administration of crude curcumin results in highly variable serum concentrations, ranging from below detection (<1 ng/mL) to approximately 3,200 ng/mL (≈3.2 µg/mL), depending on dose (2–10 g), formulation, and inter-individual factors (Dei Cas and Ghidoni 2019; Salehi et al. 2019). These values represent the upper limits of total circulating curcumin, predominantly glucuronide and sulfate conjugates, rather than free parent compound. In a crossover study in healthy humans using a micelle-based formulation (NovaSOL^®^ Curcumin) designed to enhance absorption, the highest reported peak plasma concentration of total curcumin was 4,950 nM (≈1.8 µg/mL), whereas unconjugated curcumin remained minimal and substantially below the concentrations typically required to elicit effects *in vitro* (Kroon et al. 2025). By contrast, *in vitro* studies frequently test curcumin at 2–100 µg/mL (≈5–270 µM) to induce apoptosis or inhibit proliferation, with particular emphasis on inhibitory concentrations (Dei Cas and Ghidoni 2019; Fança-Berthon et al. 2021). For instance, curcumin has been reported to inhibit proliferation and induce apoptosis in A549 lung cancer cells within this range, and to induce approximately 95% cell death in NCI-H460 large cell lung cancer cells at 30 µM (≈11.1 µg/mL), a concentration at least 10x higher than those achievable systemically in humans (Wu et al. 2010; Endo et al. 2020; Hegde et al. 2023). Furthermore, limited *in vivo* data suggest that even intermediate doses may be insufficient as monotherapy: in a lung cancer xenograft model, curcumin at 10 µM (≈3.7 µg/mL) did not significantly inhibit tumor growth, prompting evaluation in combination with epigallocatechin gallate, while dietary curcumin at 4,000 ppm was associated with lesion progression in other lung cancer models (Dance-Barnes et al. 2009). These doses reflect administered amounts rather than measured plasma levels, which remain much lower due to curcumin’s rapid metabolism, poor solubility, and limited absorption. Collectively, these findings highlight that curcumin concentrations effective in standard *in vitro* assays do not necessarily reflect plasma levels that are achievable or pharmacologically active *in vivo*.

In the present study, native curcumin was tested at 4 µg/mL and 10 µg/mL (≈10–27 µM) to investigate cellular responses at low, plasma level-informed *in vitro* concentrations selected based on reported upper ranges of total (conjugated) systemic exposure and the lower end of concentrations commonly used in anticancer *in vitro* studies. These conditions were selected as relatively low, plasma level-informed *in vitro* doses rather than as direct surrogates for clinically achievable free curcumin levels, which are considerably lower. The aim was to assess whether such concentrations produce measurable effects on viability and associated proliferation and apoptosis signaling in A549, H460, Caco-2, and HT29 cells, and to benchmark these responses against those observed at higher concentrations (20 and 50 µg/mL) typically used in conventional cytotoxic *in vitro* studies.

## Methods

### Materials

A549, NCI-H460, Caco-2, and HT29 human cancer cell lines were purchased from ATCC (Manassas, VA, USA). Dulbecco’s Modified Eagle Medium (DMEM; D5796), RPMI-1640 (R8758), L-glutamine (G7513), Trypsin-EDTA (25300-054), Phosphate Buffered Saline (D8537), penicillin-streptomycin (P4333) were purchased from Sigma-Aldrich. Fetal bovine serum (FBS, SFBSF8) was purchased from Bovogen.

Primary antibodies against Cyclin D1 (ab16663), Histone H3 (ab1791), Caspase 3 (abm1c12), β-Actin (ab8226), α-tubulin (ab4074) and Tubulin (ab59680) were purchased from Abcam and ACSL4 (22401-1-AP) and GPX4 (67763-1-Ig) were obtained from Proteintech. Secondary antibodies, anti-rabbit IRDye 800CW (926–32213), and anti-mouse 680RD (926–68072) were purchased from LI-COR Biosciences, while goat anti-rabbit Alexa Fluor^®^ 488 (ab150081) and stains, including DAPI (ab228549) and Rhodamine Phalloidin (ab235138), were purchased from Abcam. Staurosporine (S5921), Triton X-100 (T8787), paraformaldehyde (158127) and DMSO (D8418) was purchased from Sigma-Aldrich and Erastin (RDS544910) from *In Vitro* Technologies. Curcumin (95% purity, HPLC/MS authenticated) was provided by The Health Ingredients Co. (Australia); certificate of analysis provided in the Supplementary File.

MTS reagent (G3582) was purchased from Promega. Complete Mini Protease Inhibitor Cocktail Tablet was obtained from Roche; the BCA Protein Assay Kit from Thermo Fisher Scientific; 4–20% SDS-PAGE Gels from Bio-Rad; PVDF membranes from Merck; and Intercept^™^ (PBS) Blocking Buffer from LI-COR Biosciences.

### Cell lines

A549 (lung adenocarcinoma) and Caco-2 (colorectal adenocarcinoma) cells were cultured in DMEM, while NCI-H460 (large cell lung carcinoma) and HT29 (colorectal adenocarcinoma) cells were maintained in RPMI-1640. Both media were supplemented with 10% FBS, 1% L-glutamine, and 0.5% penicillin/streptomycin. Cells were sub cultured using 0.05% Trypsin-EDTA and incubated at 37 °C in a humidified atmosphere of 5% CO_2_. All experiments were conducted using cell lines between passages 3 and 20.

### Cell viability using the MTS assay

Curcumin was dissolved in DMSO to prepare a 20 mg/mL stock solution and used at final concentrations ranging from 4 to 50 µg/mL.

The MTS assay was performed using the CellTiter 96^®^ AQueous One Solution Assay Kit (G3582, Promega) according to the manufacturer’s instructions. Briefly, cells were seeded into 96-well plates at 1 × 10^4^ cells/well and incubated for 24 h to reach confluency. Cells were treated for 24 h with 4, 10, 20, and 50 µg/mL curcumin and appropriate controls, including media only, 0.05% TX100 in 1X PBS (negative control) and vehicle control where cells were treated with equivalent concentrations of DMSO.

Assay blanks included media alone and curcumin-only blanks for each concentration to account for any color interference with absorbance readings at 490 nm. All experiments were conducted in three biological replicates, each with three technical replicates. Cell viability (%) was calculated using the formula:

$$\text{Cell}\;\text{viability}\;\left(\text{\%}\right)\;=\;\frac{\left(\text{Absorbance}\;\text{of}\;\text{treatment}\;-\;\text{Absorbance}\;\text{of}\;\text{corresponding}\;\text{blank}\right)}{\left(\text{Absorbance}\;\text{of}\;\text{vehicle}\;\text{control}\;-\;\text{Absorbance}\;\text{of}\;\text{media}\;\text{blank}\right)}\;\times \;100\text{\%}$$

Cell viability was also monitored using a Nikon Eclipse Ts2 inverted microscope throughout the experiment.

### Confocal microscopy

Cells were seeded into Corning 96-well High-Content glass-bottom screening microplates at a density of 5 × 10³ cells/well. After 24 h, cells were treated with either control (media only) or 4 µg/mL curcumin. After 24 h, cells were fixed with 4% paraformaldehyde/PBS, washed 3 times with 1xPBS and permeabilized using 0.5% Triton X-100/PBS. Immunocytochemistry involved blocking nonspecific binding using 2% bovine serum albumin (BSA) in PBS for 1 h, followed by overnight incubation at 4 °C with a primary antibody against Cyclin D1 (1:1000), followed by 1-hour room temperature incubation with goat anti-rabbit Alexa Fluor 488 IgG secondary antibody, DAPI and Rhodamine Phalloidin. Images were collected using 20x objective as Z-stacks using Olympus FLUOVIEW FV3000 confocal microscope. Approximately 10–12 images per treatment group (covering ∼1000 nuclei) were captured from two independent experiments.

### Image analysis

Image analysis was performed using ImageJ v1.54g. Images were opened as hyperstacks containing 12 slices in split-channel, 24-bit composite format. Each image was separated into three individual channels: DAPI (blue), Cyclin D1 (green), and Rhodamine Phalloidin (red), then merged into a single composite while retaining the multilevel structure. All images were converted to 8-bit format, and the threshold was set to Otsu.

To determine mean fluorescence intensity (MFI) of Cyclin D1, a nuclear mask was generated using the DAPI channel. Particle analysis parameters were set to a size range of 10–infinity and circularity of 0.00–1.00. This mask was applied to the Cyclin D1 channel, and MFI was measured for all nuclei, reflecting the subcellular localization levels of Cyclin D1 within the nuclei. This nuclear cyclin D1 MFI of curcumin 4 µg/mL treatment is presented as a dot plots of each individual nucleus MFI.

### Western blotting

Cells were seeded at a density of 6 × 10^5^ per 100 mm Nunc^™^ Petri dish and cultured until confluency. Treatments included curcumin (4–50 µg/mL) for 24 h, staurosporine for 4 h (apoptosis positive control), or erastin for 48 h (ferroptosis positive control). Following treatment, cells were washed with ice-cold 1× PBS and lysed in buffer (20 mM Tris pH 7.5, 150 mM NaCl, 1 mM EDTA, 1% Triton X-100) supplemented with a cOmplete ^™^ Mini Protease inhibitor cocktail tablet. Lysates were incubated on ice for 20 min and centrifuged in microfuge at 14,800 rpm for 15 min at 4 °C. Supernatants were collected, and protein concentrations were quantified using the BCA Protein Assay Kit according to manufacturer’s instructions. Total protein concentrations were determined using 0–1.2 mg/ml BSA standard curve.

Equal amounts of protein (40 µg) were prepared in Laemmli buffer and denatured by 5 min incubation at 100 °C. The proteins were resolved on 4–20% SDS-PAGE and transferred onto PVDF membranes. Membranes were blocked with Intercept^™^ (PBS) Blocking Buffer for 1 h and incubated overnight at 4 °C with an indicated combination of the following primary antibodies against Tubulin, β-Actin, Caspase 3, ACSL4/FACL4, and GPX4, all diluted 1:1000. After three washes in 0.01% Tween-20/PBS, membranes were incubated for 1 h at room temperature with secondary antibodies—anti-rabbit 800CW or anti-mouse 680RD—both diluted 1:2000. Blots were washed again and visualized using the ChemiDoc^™^ imaging platform (Alliance Q9 Advanced, Uvitec). Band intensities were quantified using ImageJ v1.54g, and relevant protein expression was normalized to the loading controls. Western blotting was performed in biological triplicates with at least two technical replicates per cell line.

### Statistical analysis

Statistical analysis was performed using GraphPad Prism version 10. A Mann-Whitney U test following normality testing was used to compare MFI between control and 4 µg/mL curcumin-treated cells in the immunofluorescence assay. One-way ANOVA, followed by Dunnett’s post hoc test was used for the MTS and Western blotting data, with all treatments compared to the control. For ferroptosis analysis, western blotting data, Two-way ANOVA was performed. Data are presented as mean ± standard deviation (SD). All p-values were two-sided, with *p* < 0.05 considered statistically significant.

## Results

### Cell death is not observed at low, plasma level-informed curcumin concentrations

**Figure 1.**
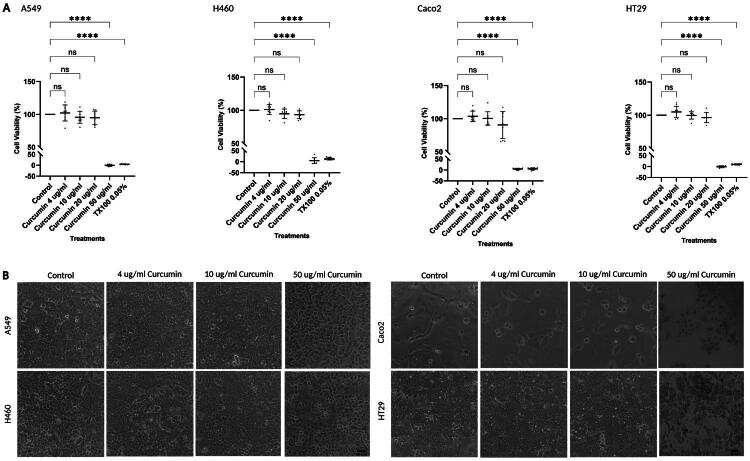
No cell death observed in all cancer cell lines at low, plasma level-informed curcumin concentrations. A549, H460, Caco-2, and HT29 cells were incubated with increasing concentrations of curcumin for 24 h. Cell viability was assessed using an MTS assay as described in the Materials and Methods section. (A) Graphs display the mean percentage cell viability for each treatment, with individual data points shown (n = 9; three independent experiments performed in technical triplicate). Error bars represent standard deviation. Statistical analysis was conducted using one-way ANOVA followed by Dunnett’s post hoc test, comparing each treatment to the control. Significance is indicated as follows: *p < 0.05, **p < 0.01, ***p < 0.001, ****p < 0.0001, ns = not significant. (B) Images represent cell morphology following curcumin treatment. Images were captured using a Nikon Eclipse Ts2 inverted microscope. Scale bar: 40 µm.

The effects of low *in vitro* concentrations of curcumin (up to 4 µg/mL), selected based on reported plasma ranges, were assessed for cytotoxicity (Dance-Barnes et al. 2009; Zhou et al. 2013; Dei Cas and Ghidoni 2019). Cell viability was measured across all lung and CRC cell lines using the MTS cell proliferation assay. At 4 µg/mL, no significant reduction in viability was observed compared to untreated controls, with mean viability values ranging from 101.1% to 104.9% (A549: 102.1% ± 12.2; H460: 101.1% ± 7.7; Caco-2: 103.6% ± 7.9; HT29: 104.9% ± 8.6; *n* = 3; *p* > 0.05) (Figure 1A). A slight decrease in viability, less than 10% was first observed at 20 µg/mL (except in Caco2 cells), though this did not reach statistical significance. These findings were supported by light microscopy, which showed no visible signs of cell death at 4–10 µg/mL, with all cell lines displaying viable cell morphology (Figure 1B).

Significant reductions in cell viability were only evident at 50 µg/mL, where reduced viability and cytotoxicity was observed: A549: −0.4% ± 4.4, H460: 4.6% ± 13.0, Caco-2: 4.3% ± 3.7, HT29: −1.5% ± 4.3 (*n* = 3; *p* < 0.0001). Correspondingly, light microscopy revealed apoptotic-like changes such as cell shrinkage and detachment in A549, H460, and Caco-2 cells. In addition, HT29 cells exhibited an accumulation of dark intracellular staining in a concentration-dependent manner as curcumin concentrations increased from 10 to 50 µg/mL.

While previous studies have reported that DMSO does not influence cell viability (Da Violante et al. 2002; Kim et al. 2013; Daker et al. 2016), the present study observed that DMSO at concentrations between 0–0.25% slightly increased cell viability across all four cell lines, with statistically significant increases observed between 0.02% and 0.25% compared to control (Refer to Supplementary File). These effects have been accounted for in cell viability calculations presented in Figure 1A. Triton X-100 (0.05%) served as the positive control, as its membrane-disrupting action reliably induces cell death, confirming the assay’s capacity to detect loss of viability.

### Low, plasma level-informed curcumin concentration (4 µg/mL) promotes Cyclin D1 translocation to nucleus

Considering the monophasic response, with cell death occurring only at higher curcumin concentrations (Figure 1), further investigation was undertaken to examine the mechanisms underlying cell survival and biological responses at lower, sub-cytotoxic concentrations.

The cell cycle is regulated by cyclins, including cyclins D1, D2, and D3, which function in association with their cyclin-dependent kinases (CDKs) (Tchakarska and Sola 2020). Cyclin D1 expression is regulated by external growth signals through activation of RAS-dependent signaling cascades, resulting in fluctuating levels and dynamic subcellular localization throughout the normal cell cycle, where cytoplasmic cyclin D1 binds to and activates the CDK4/6 complex (Yang et al. 2006). This complex then translocates to the nucleus during the early to late G1 phase, where the Cyclin D1–CDK4/6 complex facilitates the G1-to-S phase transition by promoting cell cycle progression (Tchakarska and Sola 2020). Therefore, increased nuclear Cyclin D1 levels indicate active cell cycle progression.

**Figure 2.**
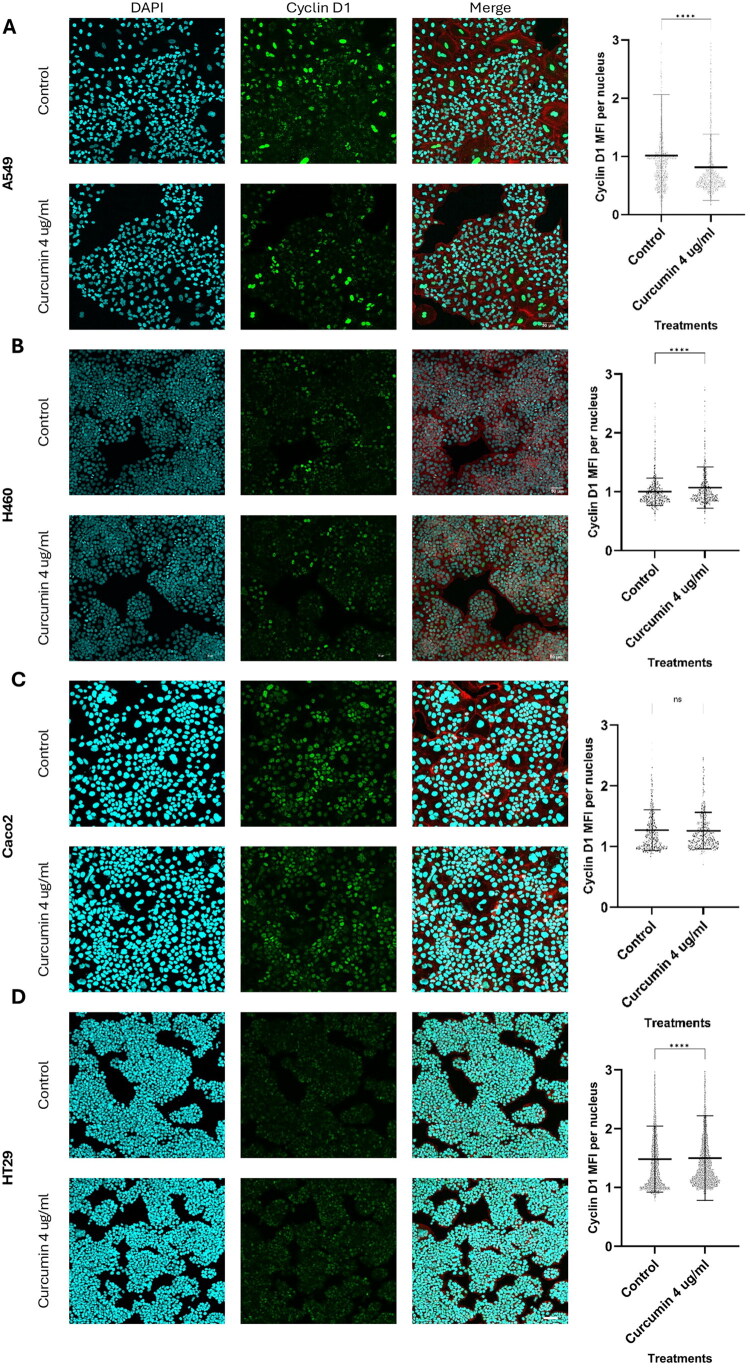
Low, plasma level-informed curcumin concentration promotes cyclin D1 translocation to the nucleus in H460, and HT29 cells. Confocal immunofluorescence images (left) and corresponding quantification of nuclear cyclin D1 levels (right) for A549 (A), H460 (B), Caco-2 (C), and HT29 (D) cells. Cells were incubated in the presence or absence of 4 µg/mL curcumin for 24 hours. After treatment, cells were fixed and incubated with a rabbit recombinant monoclonal anti-cyclin D1 antibody, followed by a goat anti-rabbit Alexa Fluor 488 secondary antibody. DAPI (blue) and Rhodamine Phalloidin (red) were used to stain nuclei and F-actin, respectively. Images were captured using an Olympus FLUOVIEW FV3000 Confocal Microscope as 1 µm slices and processed into Z-stacks in Olympus Fluoview FV31S-SW software. Brightness and contrast adjustments (LUT range: min 44, max 1915) were applied uniformly within each image panel across all treatments and cell lines for visual comparison and image display only. These adjustments did not affect the quantification, which was performed on the original, unprocessed images. No other post-processing was applied. Dot plots represent individual Cyclin D1 MFI values per nucleus from two independent biological replicates (≥1000 nuclei/treatment). Lines indicate group means; error bars represent standard deviation. Statistical comparisons were performed using the Mann–Whitney U test after normality assessment (Shapiro–Wilk test). *p < 0.05, **p < 0.01, ***p < 0.001, ****p < 0.0001, ns = not significant. Scale bar: 50 μm.

As MTS results indicated no/slight increase in cell viability across all four cell lines in the presence of 4 µg/mL curcumin compared to the control (Figure 1), immunofluorescence was performed to assess cyclin D1 localization following curcumin treatment. As shown in Figure 2, Cyclin D1 co-localised with DAPI (indicating nuclear localization) and also exhibited diffuse cytoplasmic staining, as indicated by its presence outside of the DAPI-stained nucleus but within the actin-bordered cell perimeter stained by Rhodamine phalloidin (Figure 2).

Quantification of Cyclin D1 translocation using mean fluorescence intensity (MFI) per nucleus revealed distinct responses across the cell lines. In H460 cells, the nuclear MFI of curcumin-treated cells (1.1 ± 0.4, *n* = 1713) was significantly higher than that of the control group (1.0 ± 0.2, *n* = 2018; Mann–Whitney U test, *p* < 0.001). Similarly, in HT29 cells, curcumin-treated cells showed a significantly higher nuclear MFI (*M* = 1.5 ± 0.7, *n* = 3643) compared to control (1.5 ± 0.6, *n* = 3665; *p* < 0.001). In Caco-2 cells, a similar trend was observed, with curcumin-treated cells showing comparable levels of nuclear MFI (1.3 ± 0.3, *n* = 1154) compared to control (1.3 ± 0.3, *n* = 1322), though this difference was not statistically significant (*p* = 0.17). In contrast, A549 cells treated with 4 µg/mL curcumin showed a significantly lower nuclear MFI (0.8 ± 0.6, *n* = 1909) compared to control (1.0 ± 1.0, *n* = 2640; *p* < 0.001). At 4 µg/mL curcumin, the H460 and HT29 cell lines show nuclear Cyclin D1 translocation, whereas A549 shows the opposite trend. These findings highlight heterogeneity in curcumin-induced Cyclin D1 translocation across the different cancer cell lines.

While at a cell population level (Figure 2), nuclear Cyclin D1 intensity shows cell line–dependent responses the dot plots also reveal variability in fluorescence intensity across individual nuclei. Visually, Cyclin D1 fluorescence levels varied between cells within the same image, while DAPI intensity remained relatively consistent. This variability, captured by the spread of individual data points, likely reflects differences in cell cycle stage, as nuclei of cells preparing to divide display brighter Cyclin D1 fluorescence.

### Procaspase 3 remains uncleaved at the low, plasma level-informed curcumin concentration

As cells remain viable and H460 and HT29 cells show indications of cell cycle survival and/or progression at 4 µg/mL curcumin (Figure 2), western blotting was performed to investigate cell death *via* apoptosis. This was performed by measuring total cellular levels of procaspase 3, an executioner caspase central to both intrinsic and extrinsic apoptosis ppathways(Kashyap et al. 2021). All curcumin concentrations were assessed, 4–50 µg/mL for A549, H460, and HT29 cells, and 4–20 µg/mL for Caco-2 cells (as complete cell loss at 50 µg/mL prevented protein extraction in Caco-2), to determine at which concentration procaspase 3 levels decreased, reflecting apoptosis.

**Figure 3.**
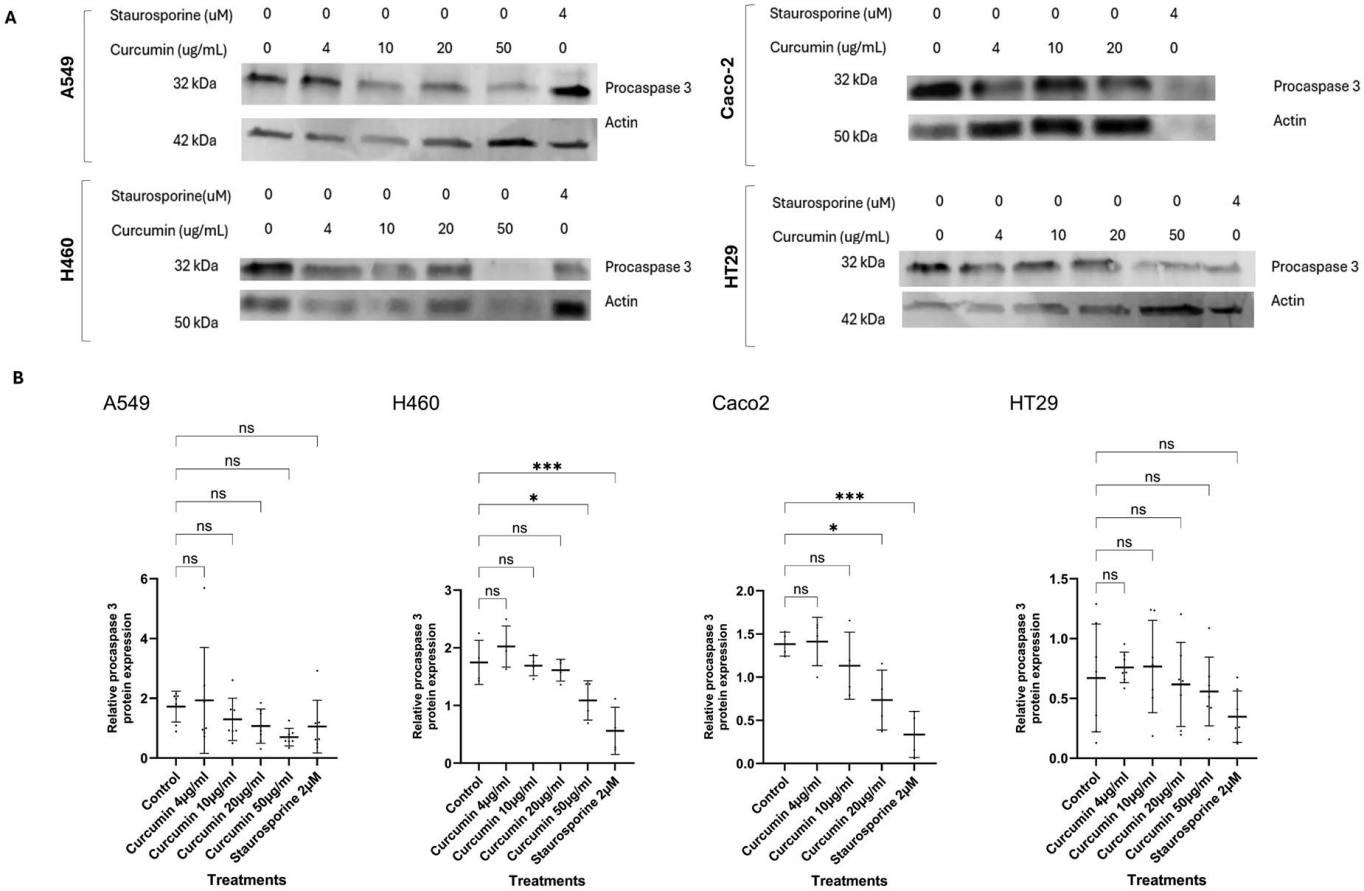
Procaspase 3 levels indicate lack of curcumin-induced apoptosis at the low, plasma level-informed curcumin concentration. (A) A549, H460, Caco-2, and HT29 cells treated with control, curcumin (4–50 µg/mL) for 24h, or staurosporine (2 µM, positive control) for 4h. Cells were washed with PBS, lysed, and processed for Western blotting as described in Materials and Methods. Each lane was loaded with 40 µg of total protein. Membranes were probed with primary antibodies against caspase 3 and either β-actin or tubulin (loading control), followed by IRDye 680 and 800-conjugated secondary antibodies. Blots were imaged using the ChemiDoc^™^ platform. (B) Quantification of procaspase 3 band intensity was normalized to β-actin and expressed relative to the control for each cell line. Dot plots represent individual values from 3 biological replicates per cell line, with additional technical replicates included for A549 and HT29 (total n = 7), and for H460 and Caco-2 (total n = 4). Horizontal lines indicate the group mean and error bars represent standard deviation. Statistical analysis was performed using one-way ANOVA followed by Dunnett’s post hoc test. *p < 0.05, **p < 0.01, ***p < 0.001, ****p < 0.0001; ns = not significant. β-actin (ab8226) was used as the primary loading control following discontinuation of the tubulin antibody (ab59680); earlier tubulin-normalized blots are presented in the Supplementary Material.

Staurosporine significantly reduced procaspase-3 levels in Caco-2 cells (0.3 ± 0.3, *n* = 4, *p* < 0.001) and H460 cells (0.6 ± 0.4, *n* = 4, *p* < 0.001) compared to control (Caco-2: 1.4 ± 0.01, H460:1.8 ± 0.4, *n* = 4). Although not statistically significant, reductions were also observed in HT29 (0.3 ± 0.2, *n* = 7, *p* = 0.23), and A549 cells (1.1 ± 0.9, *n* = 7, *p* = 0.53) compared to control (HT29: 0.7 ± 0.5, A549:1.7 ± 0.5, *n* = 7). This data indicates differential apoptotic responses across the cell lines (Figure 3).

In Caco-2 cells, curcumin at 4 µg/mL caused no change in procaspase 3 levels (1.4 ± 0.3, *n* = 4, *p* > 0.05) compared to control (1.4 ± 0.1, *n* = 4), with a significant reduction observed only at 20 µg/mL (0.7 ± 0.3, *n* = 4, *p* < 0.05). A similar trend was seen in H460 cells, where 4 µg/mL induced no change in procaspase 3 levels (2.0 ± 0.4, *n* = 4, *p* > 0.05), and a significant reduction occurred only at 50 µg/mL (1.1 ± 0.3, *n* = 4, *p* < 0.05). These results align with MTS findings, indicating relative resistance to cell death at low curcumin concentrations in these two cell lines.

HT29 cells also showed no change in procaspase 3 levels at 4 µg/mL (0.8 ± 0.1, *n* = 7) or intermediate concentrations, with a reduction evident only at 50 µg/mL (0.6 ± 0.3, *n* = 7, *p* = 0.95) compared to control (0.7 ± 0.5, *n* = 7), suggesting a higher threshold for apoptosis induction. In contrast, A549 cells showed a concentration dependent reduction in procaspase 3 from 10 µg/mL, with a marked decrease at 50 µg/mL (0.7 ± 0.3, *n* = 7, *p* = 0.17), although this was not statistically significant. This suggests greater apoptotic sensitivity in A549 cells to curcumin despite their resistance to staurosporine.

Overall, statistically significant reductions in procaspase 3 occurred at higher concentrations in Caco2 and H460 cells, with nonsignificant but similar reductions in HT29 and A549 cells. Collectively, these findings confirm that the low, plasma level-informed curcumin concentration (4 µg/mL) do not consistently induce procaspase 3 cleavage across all tested cell lines, highlighting cell-line specific differences in sensitivity to curcumin-induced apoptosis.

### Curcumin-induced cell death is not mediated by ferroptosis in HT29 cells

Cell death can occur through several regulated pathways, including apoptosis, necrosis, and ferroptosis (Brockmueller et al. 2024). Ferroptosis, recently recognized as a cell death mechanism specific for colon cells, is uniquely characterized by its dependence on iron and lipid peroxidation, distinguishing it from the caspase-mediated mechanisms of apoptosis and the kinase-driven processes of necroptosis (Yang et al. 2022). It is triggered by the accumulation of lipid peroxides resulting from impaired antioxidant defenses, particularly dysfunction of the glutathione (GSH)/GPX4 axis (Yang et al. 2022; Liu et al. 2024). Iron overload, mediated through transferrin receptor 1, further contributes by inducing the Fenton reaction and generating reactive oxygen species (ROS) that oxidize polyunsaturated fatty acids (PUFAs) in cellular membranes, often observed as black staining of affected cells (Liu et al. 2024). Emerging evidence suggests that ferroptosis may play an important role in the development and progression of CRC (Liang et al. 2023; Song et al. 2023; Wu et al. 2023).

As HT29 cells showed no change in procaspase 3 levels at the low, plasma level-informed curcumin concentration (4 µg/mL) or intermediate concentrations of curcumin, with a reduction observed only at 50 µg/mL, and given their colorectal origin and the earlier observation of black staining (Figure 1), ferroptosis was investigated as a potential mode of cell death in this cell line. Cells were treated with either control media or erastin, a known ferroptosis inducer. HT29 cells showed a significant increase in ACSL4 expression (*M* = 3.2 ± 0.5, *n* = 3, *p* < 0.001) and a reduction in GPX4 expression (*M* = 0.4 ± 0.4, *n* = 3, *p* > 0.05) in the presence of erastin compared to controls (ACSL4: *M* = 1.2 ± 0.8; GPX4: *M* = 0.5 ± 0.5), suggesting that ferroptosis may be induced in this cell line.

**Figure 4.**
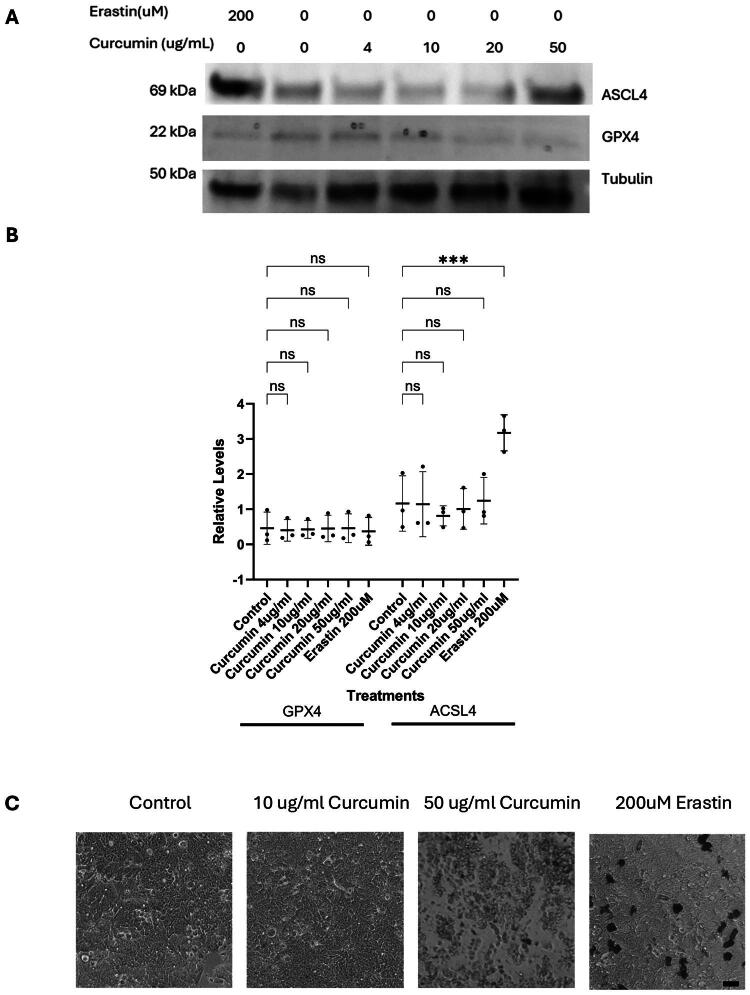
Curcumin does not induce ferroptosis in HT29 CRC cells. (A) Representative Western blot images of GPX4 and ACSL4 protein levels in HT29 cells treated with control, curcumin (4–50 µg/mL) for 24h, or erastin (200 µM, ferroptosis inducer) for 48 h. β-actin was used as a loading control. Each lane was loaded with 40 µg of total protein. (B) Quantification of GPX4 and ACSL4 protein levels across treatments. Data are presented as mean ± SD of three biological replicates. Each dot represents an individual replicate, and error bars show standard deviation. Statistical analysis was performed using two-way ANOVA followed by Dunnett’s post hoc test comparing each treatment to the control (***p < 0.001, ns = not significant). (C) Images represent cell morphology following curcumin treatment. Images were captured using a Nikon Eclipse Ts2 inverted microscope. Scale bar: 40 µm.

Based on these findings, further investigations were conducted in HT29 cells, across all curcumin concentrations (0, 4, 10, 20, and 50 µg/mL). As shown in the representative Western blot (Figure 4A), curcumin at lower concentrations (4–10 µg/mL) appeared to increase GPX4 levels relative to ACSL4, whereas at the highest concentration (50 µg/mL), GPX4 levels were reduced while ACSL4 remained elevated, similar to the GPX4 expression pattern seen with erastin. However, although these trends were visually apparent, the quantification of GPX4 and ACSL4 levels (Figure 4B) revealed high variability across replicates, and no statistically significant changes or trends were observed. Therefore, we conclude that ferroptosis pathway is not induced in HT29 cells at any curcumin concentration despite observable black staining.

## Discussion

This study evaluated the effects of curcumin at 4 µg/mL, a low *in vitro* concentration selected based on reported plasma ranges, on cell viability and apoptosis in two NSCLC lines (A549, H460) and two CRC cell lines (Caco-2, HT29). At this concentration, no statistically significant reduction in cell viability was observed in any cell line by MTS assay. In contrast, nuclear Cyclin D1 expression increased significantly in H460 and HT29 cells, suggesting maintained or enhanced cell cycle progression, while Caco-2 cells showed no change. Greater variability and high-intensity nuclei in H460, Caco-2, and HT29 cells indicate heterogeneity in Cyclin D1 levels, consistent with asynchronous cycling or subpopulations undergoing G1/S transition. Conversely, curcumin-treated A549 cells exhibited reduced nuclear Cyclin D1 and lower variability, suggesting suppression of cell cycle progression. Western blot analysis revealed no significant changes in procaspase 3 expression at 4–10 µg/mL in Caco-2 or at 4–50 µg/mL in A549, HT29, and H460 cells, indicating no apoptosis activation at these concentrations. A significant reduction in procaspase 3 was observed only in Caco-2 cells at 20 µg/mL, while reductions at 50 µg/mL in A549, H460, and HT29 were not statistically significant. Collectively, these findings demonstrate that curcumin at low, plasma level-informed *in vitro* concentrations does not induce cytotoxicity in these models and may instead modulate proliferative signaling in a cell line–dependent manner.

Among the cell lines tested, A549 cells exhibited a distinct response to curcumin, with a significant reduction in nuclear Cyclin D1 at 4 µg/mL, suggesting early cell cycle disruption. A549 cells, a human lung adenocarcinoma model harboring oncogenic KRAS while retaining wild-type EGFR and TP53, are known to be sensitive to cell cycle disruption (Zhao et al. 2008; Zhang et al. 2012; Korrodi-Gregório et al. 2016; Eng et al. 2021). Higher concentrations of curcumin have previously been shown to impair G1-to-S progression in this line through modulation of Cyclin D1 and Wnt/β-catenin signaling (Wang et al. 2018). In the present study, curcumin at 4 µg/mL reduced nuclear Cyclin D1 and altered its subcellular distribution, accompanied by increased cell-to-cell variability, consistent with dysregulated cell cycle control. Although procaspase 3 levels decreased in a concentration-dependent manner, this did not translate to reduced viability at 4 µg/mL, indicating that early apoptotic signaling or cell cycle disruption may occur without full apoptosis induction (Fallon 1990; Rasouly and Lazarovici 1994; Kabir et al. 2002). Staurosporine, a broad-spectrum kinase inhibitor that induces apoptosis primarily through disruption of tyrosine kinase signaling and G2/M arrest (Belmokhtar et al. 2001), resulted in limited apoptotic changes in A549 cells under the conditions tested. This relative resistance has been reported in other models such as L1210/0 cells and may reflect activation of caspase-independent pathways or intrinsic survival signaling (Qiao et al. 1996; Belmokhtar et al. 2001). Compared with staurosporine, curcumin demonstrated a more graded, concentration-dependent modulation of procaspase-3 in A549 cells, supporting the interpretation that low-dose curcumin primarily alters signaling dynamics rather than inducing robust cytotoxicity.

In contrast to A549, H460 cells represent an aggressive NSCLC model derived from the pleural effusion of a patient with large cell lung carcinoma, a subtype accounting for approximately 10% of lung cancers (Wang et al. 2011; American Cancer Society 2024). The presence of malignant cells in pleural fluid reflects metastatic (stage IV) disease, supporting the relevance of H460 as a model of advanced NSCLC (Patil et al. 2016). This line exhibits upregulated PI3K and TGFβ signaling pathways (Uhlén et al. 2015; Human Protein Atlas, n.d.), which promote proliferation and epithelial–mesenchymal transition (EMT) (Heavey et al. 2018), consistent with its metastatic phenotype. At 4 µg/mL, curcumin did not induce apoptosis in H460 cells. Instead, increased nuclear localization and synchronization of Cyclin D1 were observed, suggesting maintained or coordinated cell-cycle progression. Procaspase 3 levels remained unchanged at 4–20 µg/mL, with significant cell death detected only at 50 µg/mL. In contrast, 2 µM staurosporine induced clear apoptotic effects, indicating that H460 cells remain susceptible death *via* tyrosine kinase–mediated pathways. Large cell lung cancers are known for their resistance to standard treatments due to genetic mutations, tumor microenvironment changes, and activation of compensatory survival or immune evasion pathways (Shanker et al. 2010; Kang et al. 2024). Consequently, patients often experience only a brief remission following treatment and have a high likelihood of relapse, even with an initial response (Ashrafi et al. 2022). These characteristics, together with the metastatic phenotype, may contribute to the curcumin resistance observed in H460 cells, raising concerns that in highly metastatic or treatment resistant subtypes, most curcumin concentrations may fail to exert therapeutic benefit and could potentially support tumor cell survival and growth.

Caco-2 cells exhibited a clear dose-dependent response and were the most sensitive to apoptotic signaling, with significant cell death observed at 20 µg/mL curcumin and following staurosporine treatment. Nuclear Cyclin D1 levels remained unchanged at lower concentrations, while reduction in procaspase 3 and associated morphological changes at 20 µg/mL indicate apoptosis induction at higher doses. Although derived from colorectal adenocarcinoma, Caco-2 cells are widely used as a model of the intestinal epithelial barrier due to their ability to form polarized monolayers with tight junctions and active transport systems (Sonia and Sharma 2014). At lower curcumin concentrations (10 µM ≈ 3.7 µg/mL), barrier-enhancing effects have been reported, including upregulation of tight junction proteins, reduced ER stress, and decreased apoptosis (Zhou et al. 2021; Balaji et al. 2025). In contrast, at 20 µg/mL, the observed reduction in procaspase 3 suggests apoptosis induction and potential compromise of epithelial integrity. These findings support a concentration-dependent shift from a ‘tight barrier’ phenotype at low curcumin doses, consistent with its reported protective effects in inflammatory bowel disease and leaky gut syndrome (Balaji et al. 2025), to increased cytotoxicity at higher doses. In the context of colorectal cancer, disruption of barrier function could contribute to gastrointestinal side effects such as diarrhea or altered gut permeability (Panknin et al. 2023). Together, these observations highlight the importance of dose optimization in CRC, where preservation of epithelial integrity at low concentrations must be balanced against the limited cytotoxic activity observed at these levels.

HT29 cells displayed concentration-dependent changes suggestive of apoptotic responses to curcumin at higher concentrations, however, these effects were not statistically significant in the quantification. The HT29 human colorectal adenocarcinoma cell line, derived from the large intestine, exhibits characteristics of mature intestinal cells and expresses oncogenes such as *K-RAS*, *H-RAS*, *N-RAS*, *c-myc*, *Myb*, *fos* and *sis* (National Center for Biotechnology Information 2002; Verhoeckx et al. 2015; Ghodousi-Dehnavi et al. 2021). This cell line shows significant downregulation of tumor suppressors like TGF-β and TNF-α, contributing to apoptosis resistance, while MAPK pathways are hyperactivated, driving proliferation (Uhlén et al. 2015; Human Protein Atlas, n.d.). Consistent with this inherent resistance profile, no statistically significant reductions in cell viability were observed in HT29 cells at 4 µg/mL (≈10–11 µM), as assessed by MTS assay following 24 h exposure. Similarly, no significant changes in procaspase 3 levels were detected by western blot at this concentration, indicating preserved viability rather than apoptotic activation. While several studies report reduced viability in HT29 cells at comparable concentrations, reported responses are heterogeneous and highly context dependent. In many cases, reductions at 10–20 µM are modest (approximately 20–30%) or require longer exposure durations (48–72 h), with reported IC_50_ values ranging from approximately 12.9 to 50 µM depending on assay type (MTS vs. MTT/CCK-8), treatment duration, curcumin formulation, stability in culture conditions, and cell passage number (Singh et al. 2009; Wang et al. 2009; Sankpal et al. 2016). More robust activation of caspase-3 and −12 in HT29 cells has generally been observed at concentrations ≥50 µM (≈18.4 µg/mL) (Song et al. 2005). Notably in our study, HT29 cells in presence of 4 µg/mL curcumin, showed a statistically significant increase in nuclear Cyclin D1 by immunofluorescence, potentially driven by activation of the MKK3/p38δ MAPK pathway, a pro-survival mechanism previously reported in this KRAS-active cell line in response to stressors like 5-fluorouracil concentrations (Stramucci et al. 2019). This observation suggests a mechanistic basis for the absence of viability reduction at low, plasma level-informed concentrations. It is also important to recognize that although curcumin is poorly absorbed systemically, high luminal concentrations may accumulate in the gastrointestinal tract after oral administration, placing it in direct contact with the gut microbiota (Servida et al. 2024). Curcumin can modulate microbial composition, while microbial enzymes convert it into metabolites such as dihydrocurcumin, tetrahydrocurcumin, and conjugated derivatives (Scazzocchio et al. 2020; Servida et al. 2024), some of which may exhibit enhanced biological activity (Servida et al. 2024). This may partly explain its effects in intestinal epithelial cells despite limited systemic availability.

Overall, curcumin exerted cell line–specific and dose-dependent effects in both CRC and NSCLC models. A549 cells showed early susceptibility, with reduced nuclear Cyclin D1 and concentration-dependent decreases in procaspase-3. In contrast, at 4 µg/mL, curcumin did not significantly reduce viability in H460 or HT29 cells and was associated with increased nuclear Cyclin D1, suggesting maintained cell-cycle activity. Apoptotic responses were observed at higher concentrations, although statistically significant effects were confirmed only in Caco-2 and H460 cells. These findings highlight the molecular heterogeneity of the models tested and emphasize the importance of tumor-specific context when evaluating curcumin’s therapeutic potential.

### Limitations

This study’s findings derive from *in vitro* cell line models, which may not fully capture *in vivo* tumor complexity. We used native, unformulated curcumin, which may differ from the modified forms used for human administration or conjugated absorbed forms. Apoptosis assessment relied primarily on procaspase 3 levels without complementary markers (cleaved caspase-3, PARP cleavage, Annexin V/PI), limiting definitive exclusion of apoptosis; attempts to quantify cleaved caspase-3 were unsuccessful due to low expression levels. Finally, use of a single 24 h time point limits assessment of dynamic, time-dependent cellular responses.

## Conclusions

In conclusion, this study demonstrates concentration-dependent effects of curcumin on NSCLC and CRC cell lines. At low, plasma level-informed concentrations (4 µg/mL), curcumin failed to reduce cell viability or induce apoptosis, while increasing nuclear Cyclin D1, a marker of cell-cycle modulation. Significant procaspase 3 reduction was observed only in Caco-2 cells at 20 µg/mL and H460 cells at 50 µg/mL, suggesting these two lines may be modestly more susceptible to curcumin induced apoptosis. These findings indicate that curcumin does not induce cytotoxicity or robust apoptosis at low, plasma level-informed *in vitro* concentrations in these models, highlighting the need for dose optimization and cell-line specific molecular profiling to evaluate its potential adjunctive role.

## Supplementary Material

Supplemental Material

## Data Availability

The data supporting the findings of this study are available in the supplementary material provided with this article.
