# Supplementary material for: Low, plasma level‑informed native curcumin concentrations fail to induce cell death in human lung and colorectal cancer cells
Source: Pharm Biol. 2026 Mar 19;64(1):471–86. doi: 10.1080/13880209.2026.2640678 (PMC13007458; doi:10.1080/13880209.2026.2640678)

MANUSCRIPT

Figure 3_A549_Actin and caspase 3

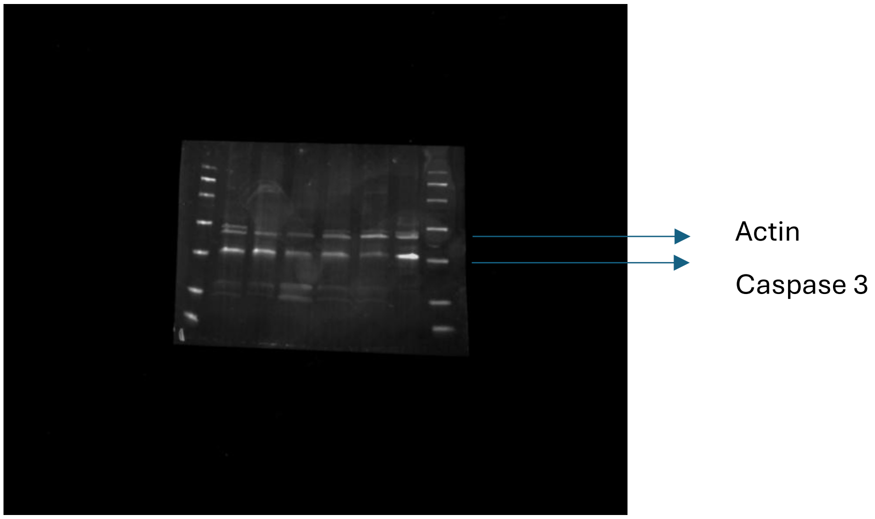

Figure 3_Caco2_Actin and caspase 3

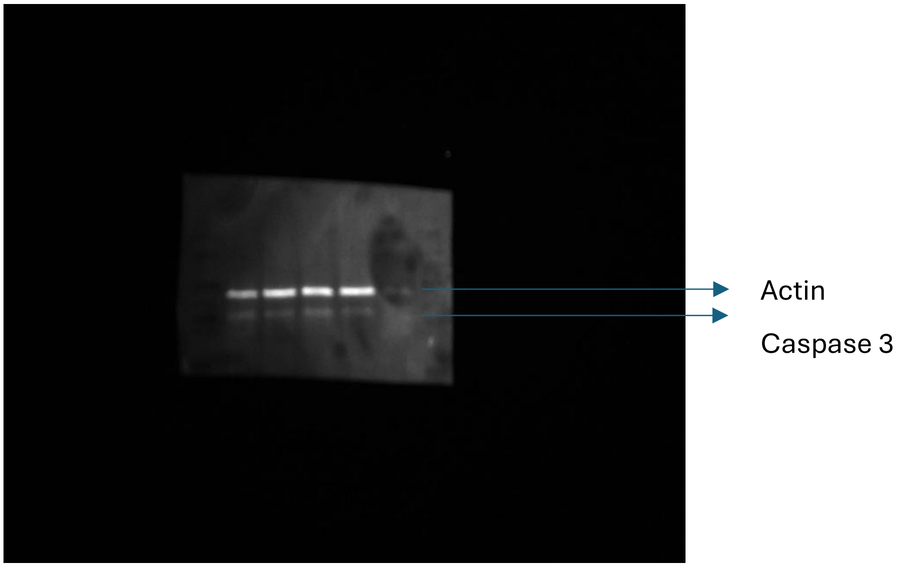

Figure 3_H460_Actin

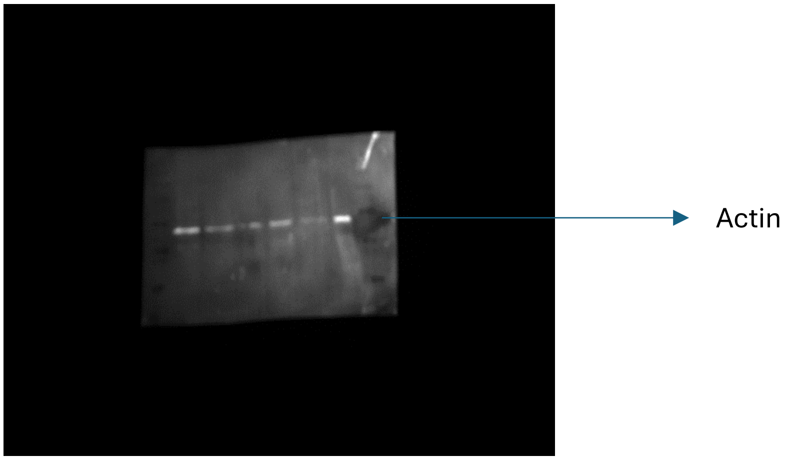

Figure 3_H460_caspase 3

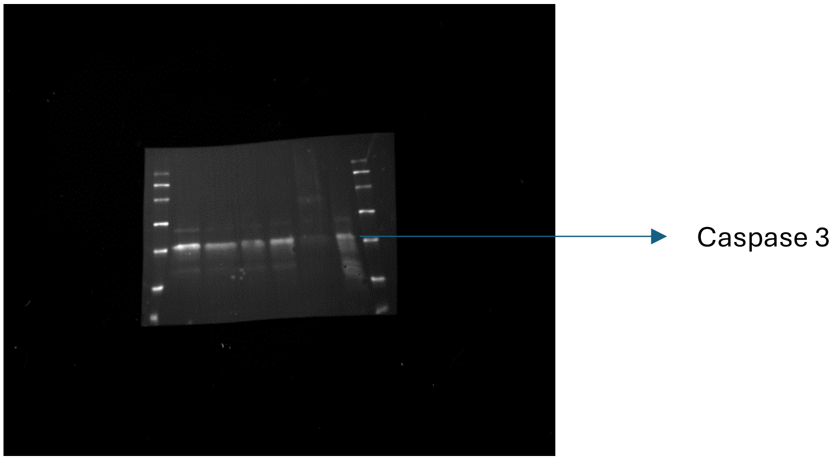

Figure 3_HT29_Actin and caspase 3

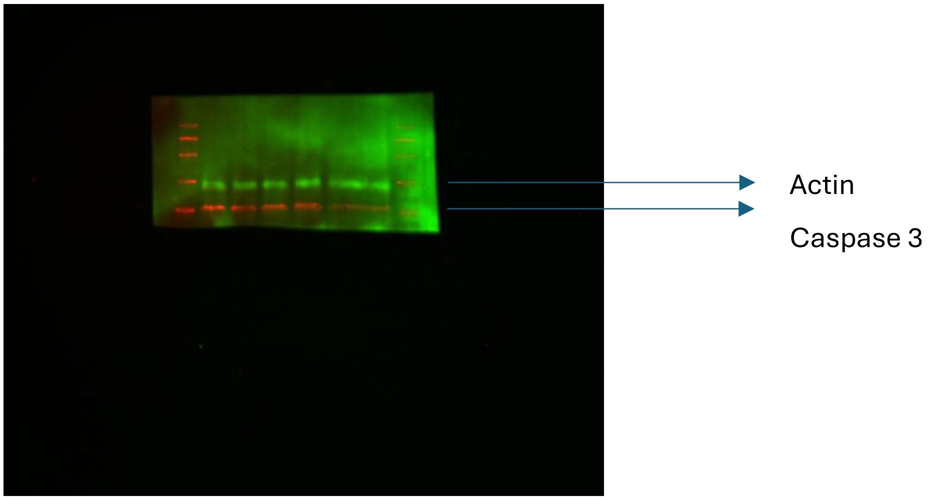

Fig 4_HT29_ ACSL4

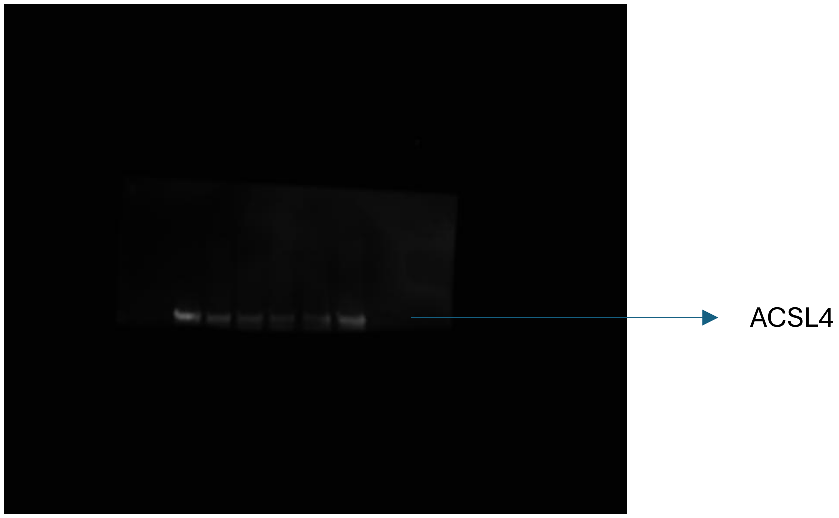

Fig 4_HT29_ GPX4 and tubulin

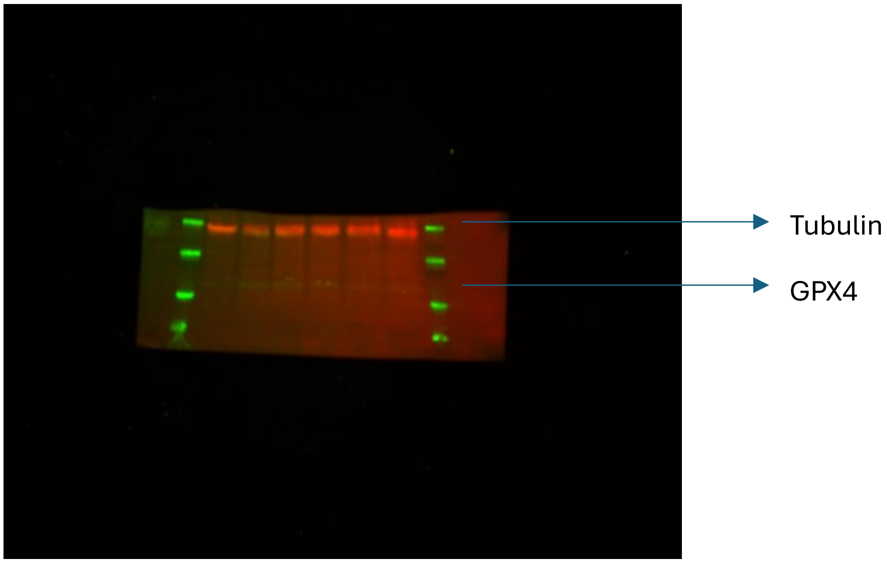

Ladder: Chameleon Duo Pre-stained Protein Ladder (Catalogue no: 928-60001) from LicorBio

SUPPLEMENTARY FILE

A549 Apoptosis

Biological replicate 1_Actin and caspase 3

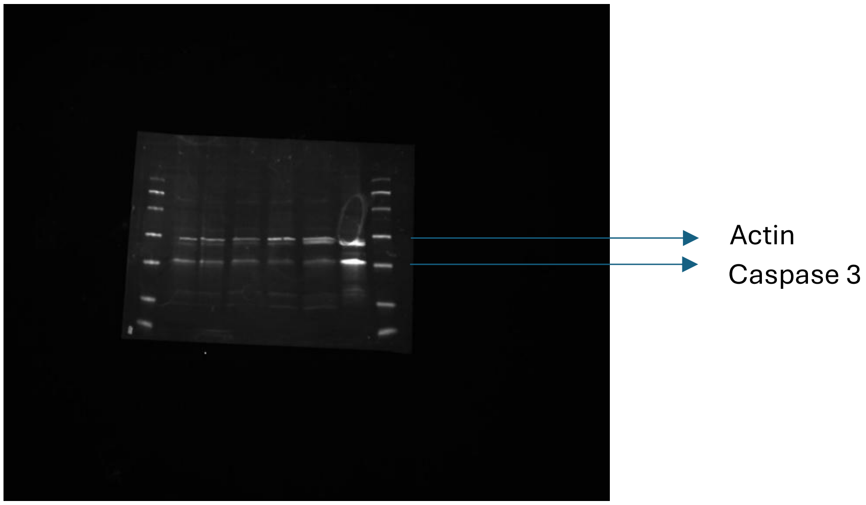

Biological replicate 2_Actin and caspase 3

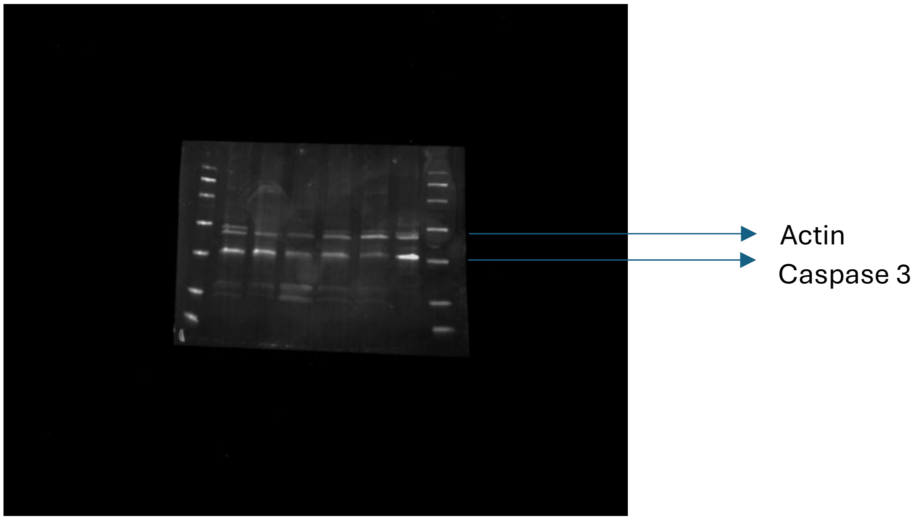

Biological replicate 3_Actin and caspase 3

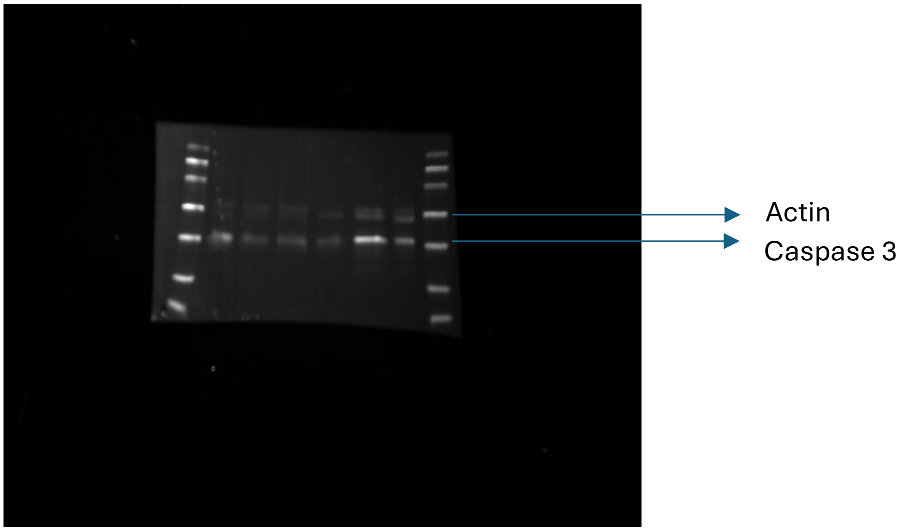

Technical replicate 1_ actin

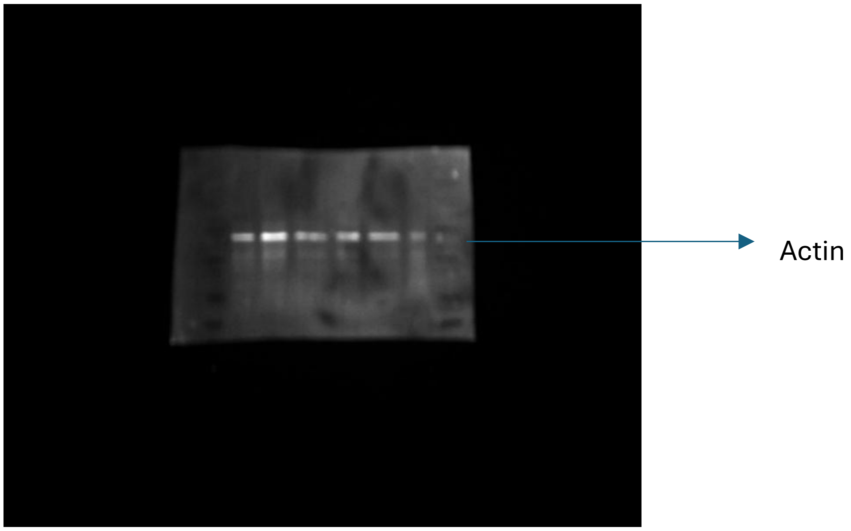

Technical replicate 1_ caspase 3

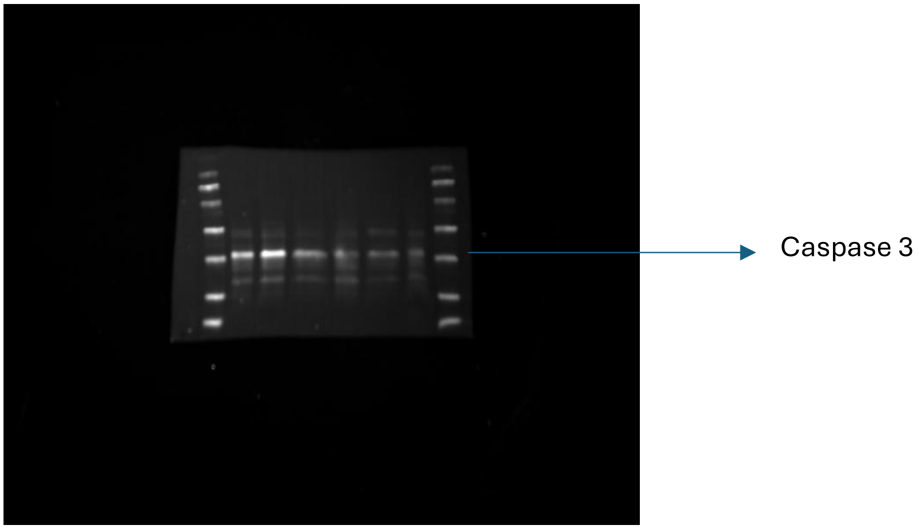

Technical replicate 2_ actin

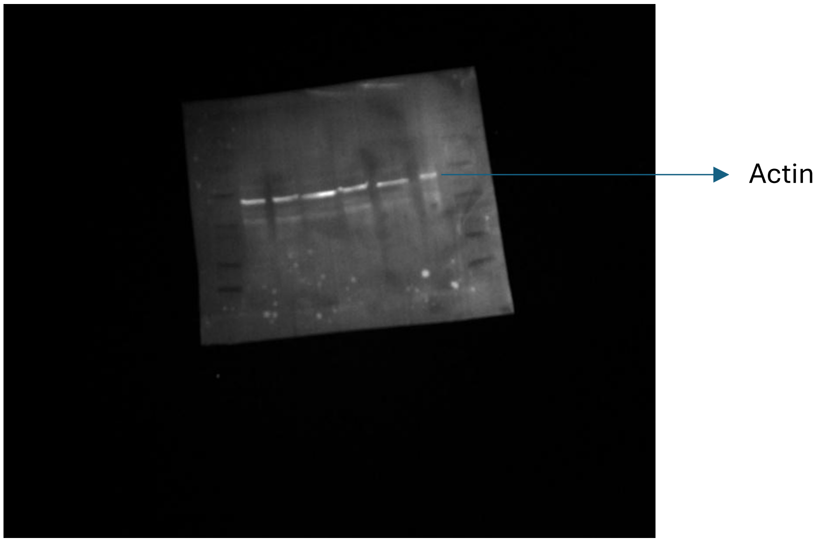

Technical replicate 2_ caspase 3

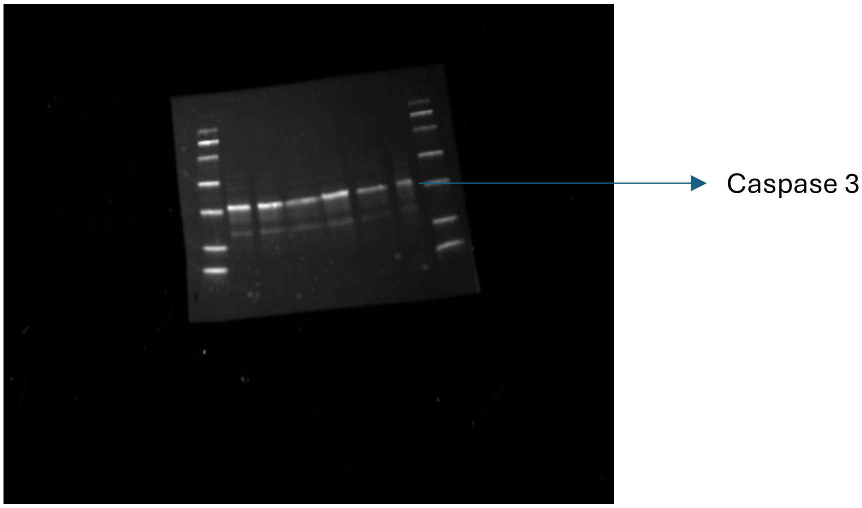

Technical replicate 3_ actin

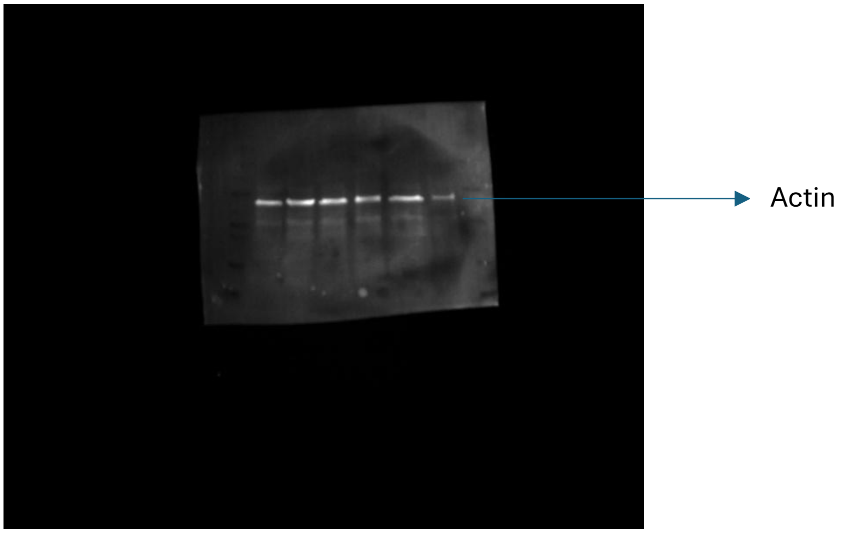

Technical replicate 3_ caspase 3

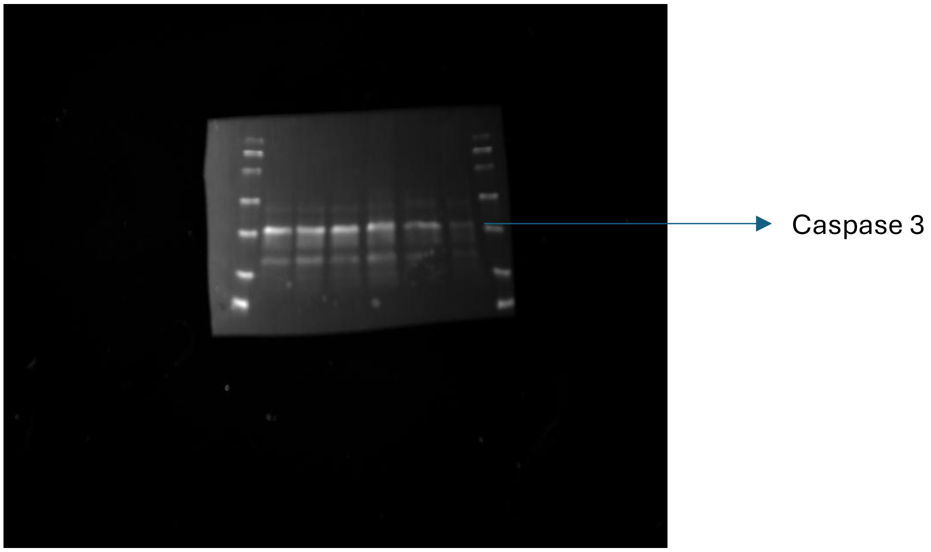

Technical replicate 4_ actin

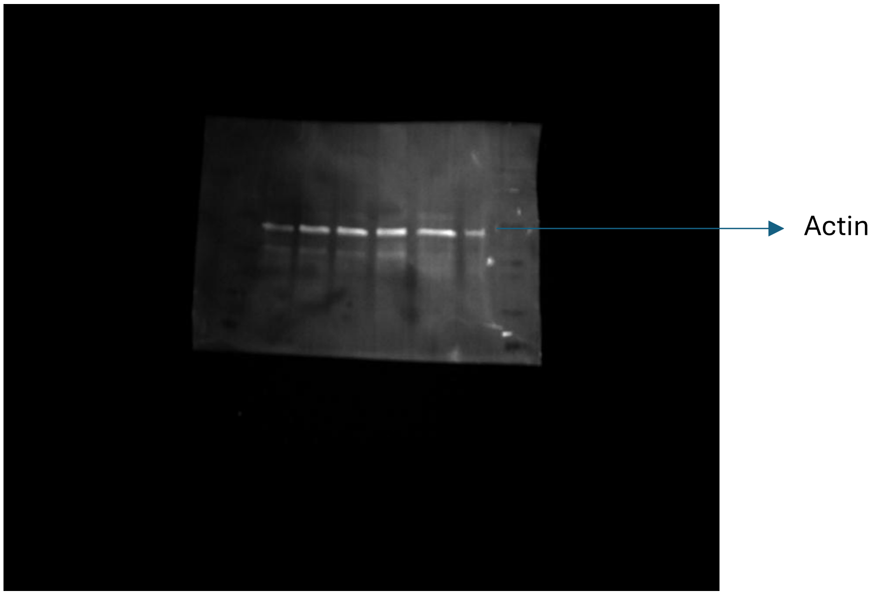

Technical replicate 4_caspase 3

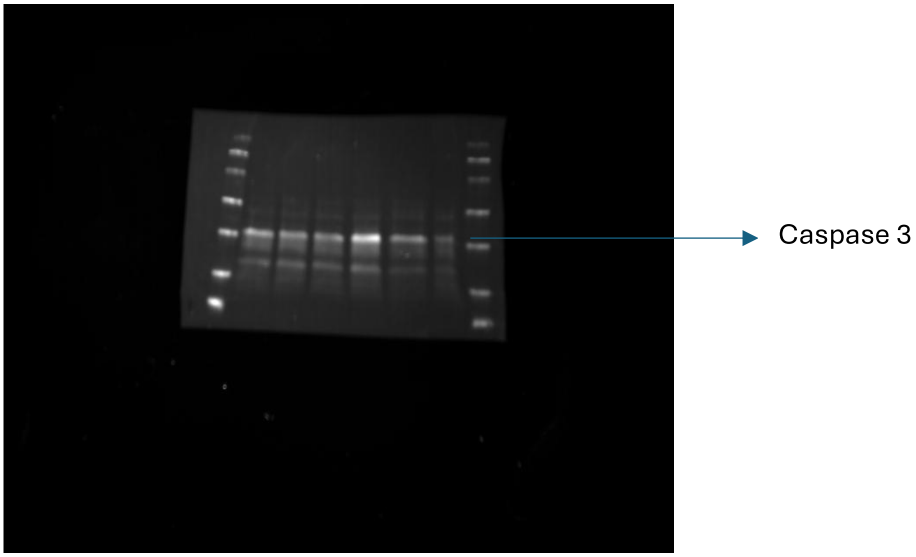

Caco2 Apoptosis

Biological replicate 1_tubulin and caspase 3

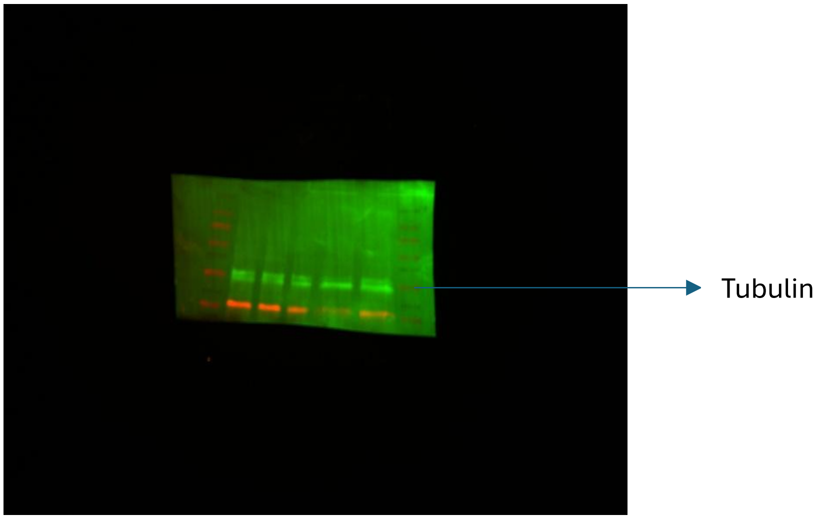

Biological replicate 2_tubulin and caspase 3

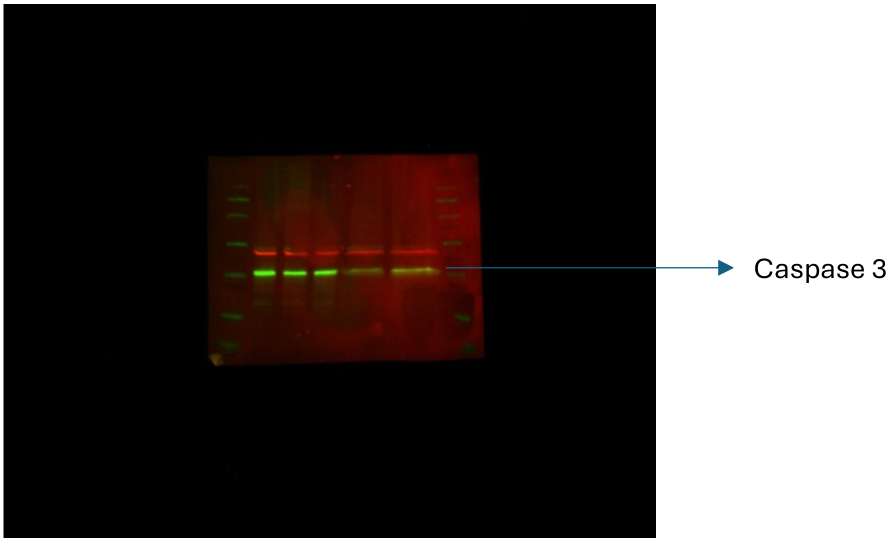

Biological replicate 3_tubulin and caspase 3

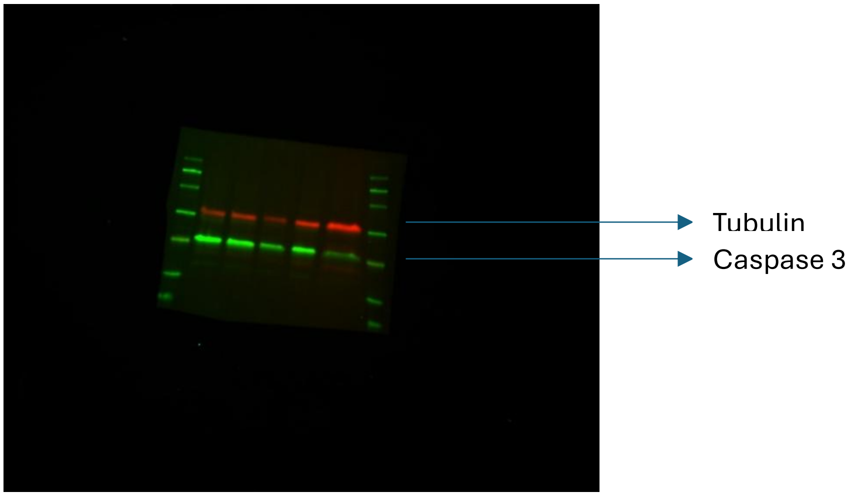

Technical replicate 1_actin

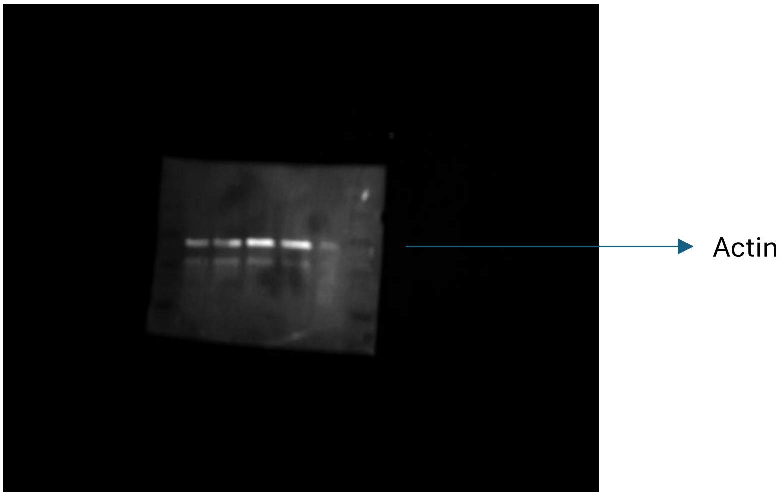

Technical replicate 1_caspase 3

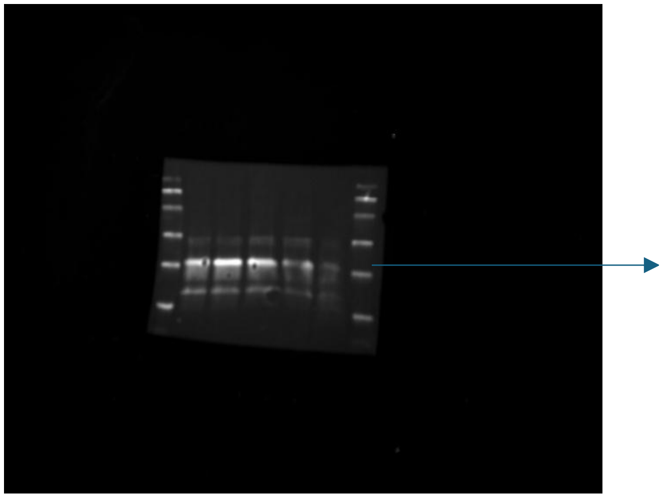

Caspase 3

Technical replicate 2_actin

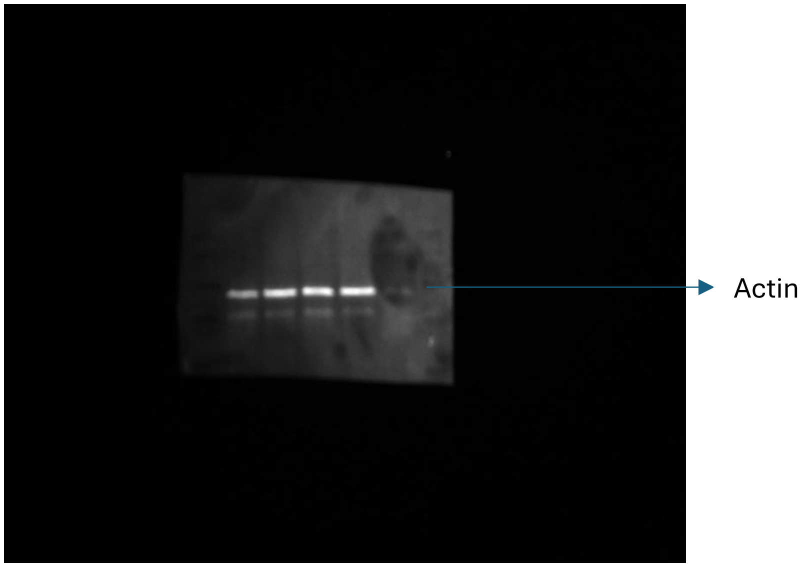

Actin

Technical replicate 2_caspase 3

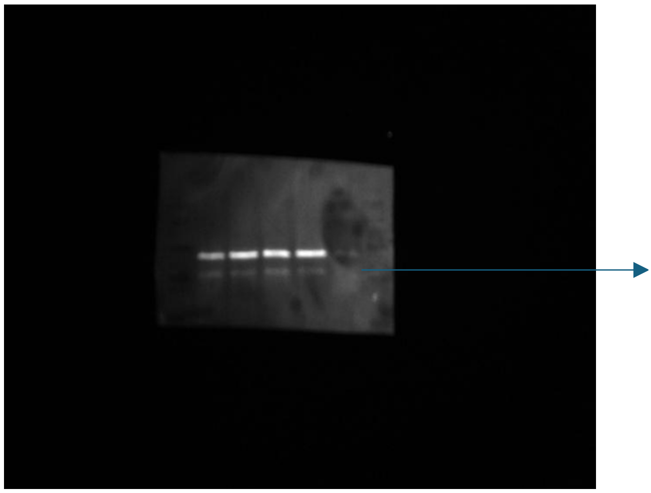

Caspase 3

Technical replicate 3_actin

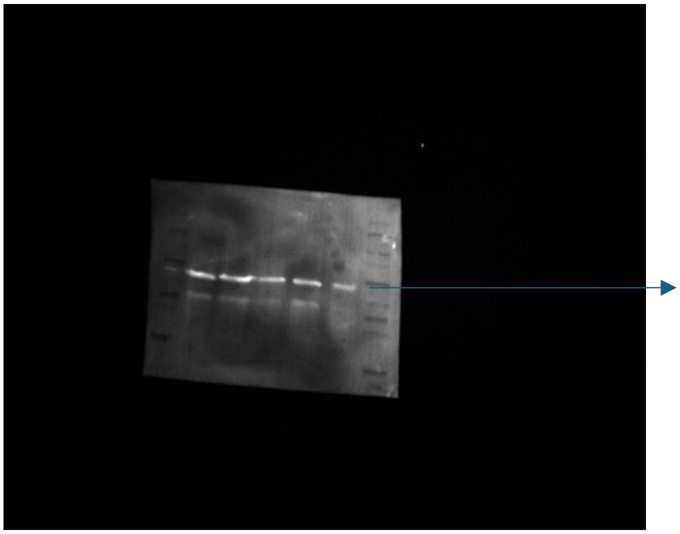

Actin

Technical replicate 3_caspase 3

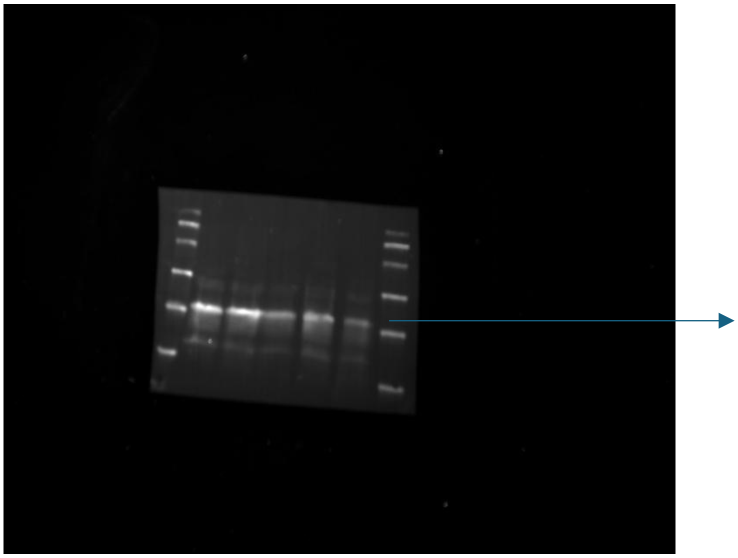

Caspase 3

Technical replicate 4_actin

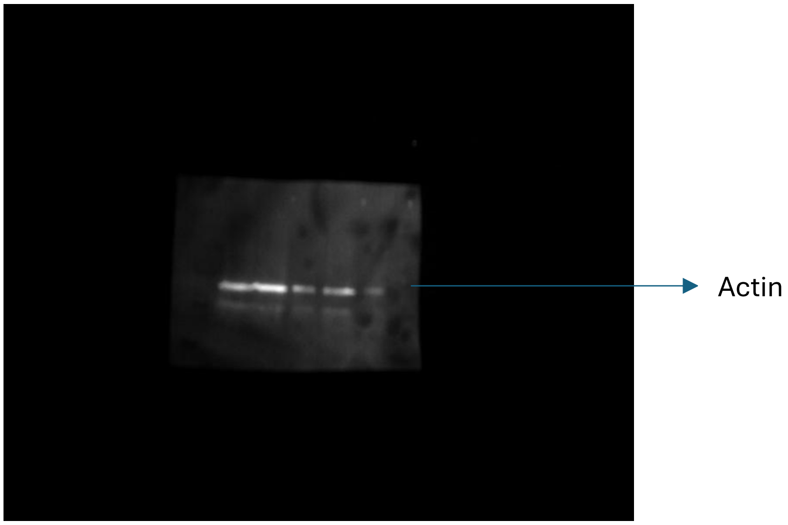

Technical replicate 4_caspase 3

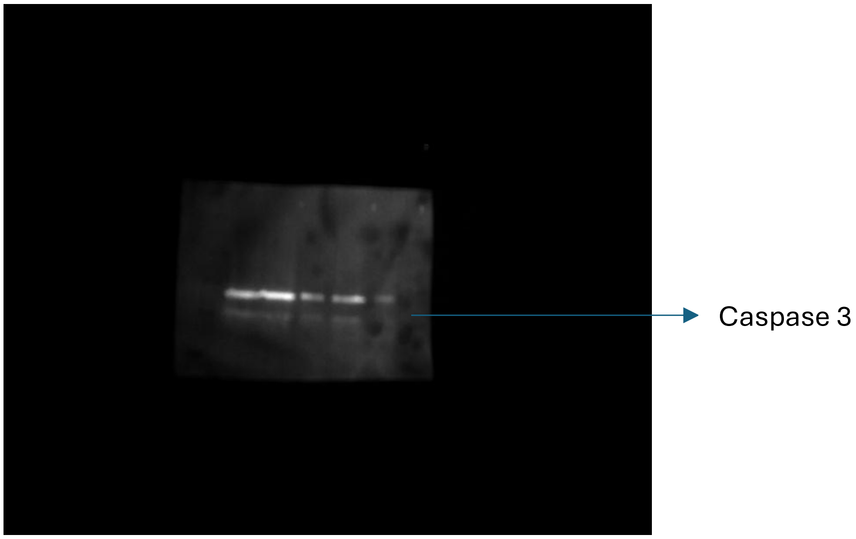

H460 Apoptosis

Biological replicate 1_tubulin and caspase 3

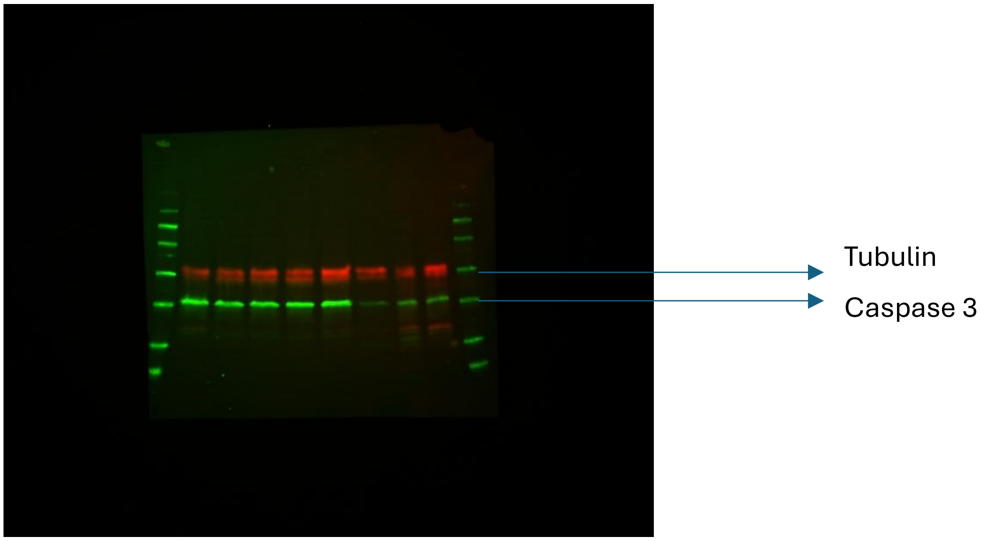

Biological replicate 2_tubulin and caspase 3

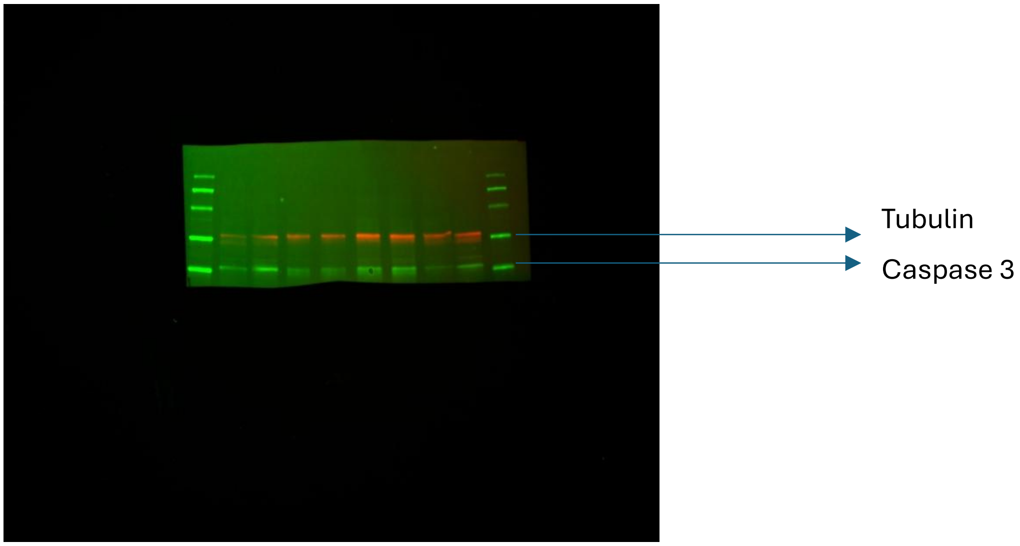

Biological replicate 3_tubulin and caspase 3

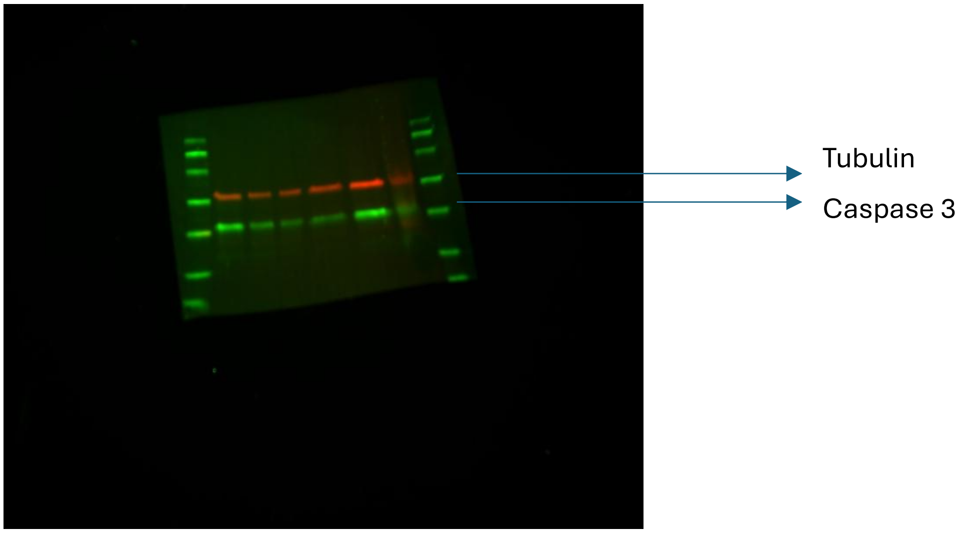

Technical replicate 1_actin

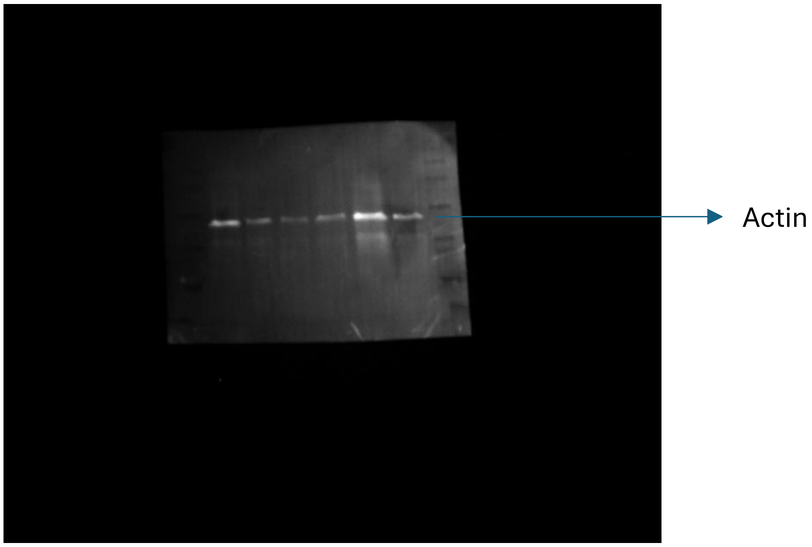

Technical replicate 1_caspase 3

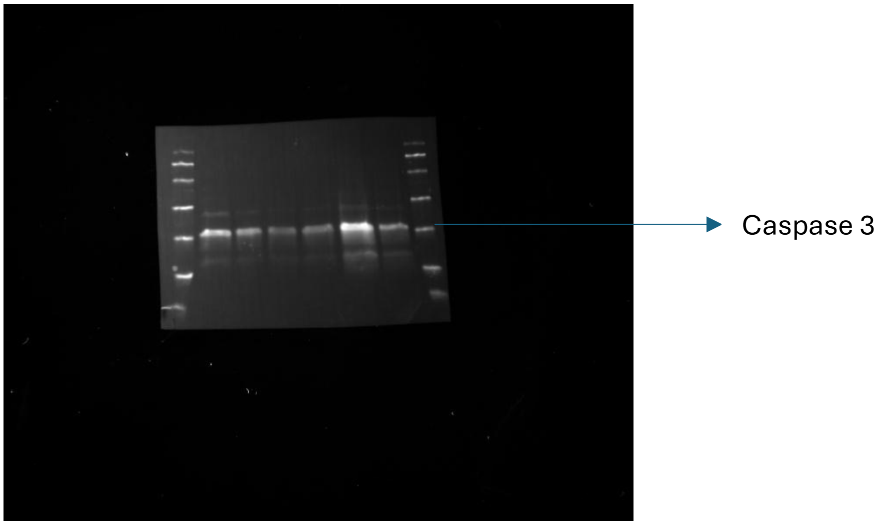

Technical replicate 2_actin

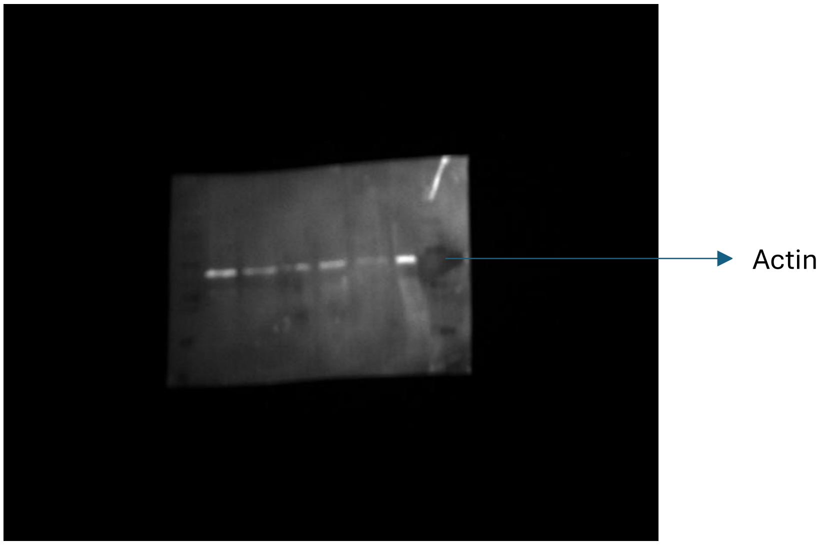

Technical replicate 2_caspase 3

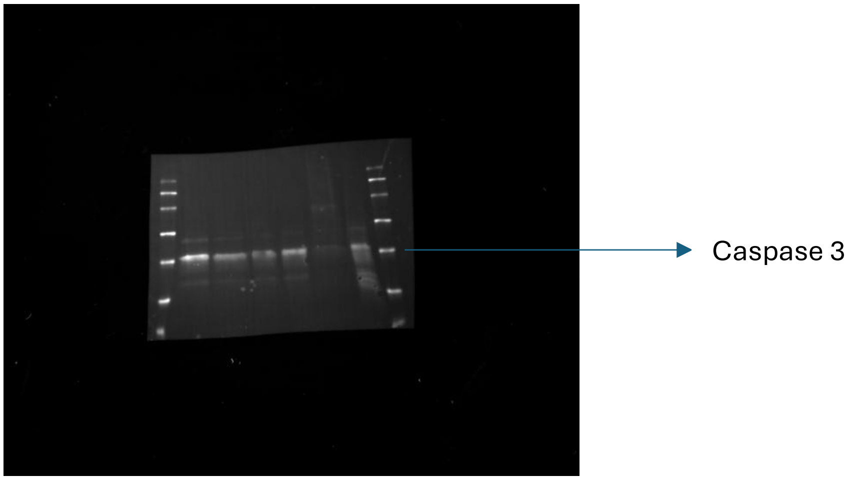

Technical replicate 3_actin

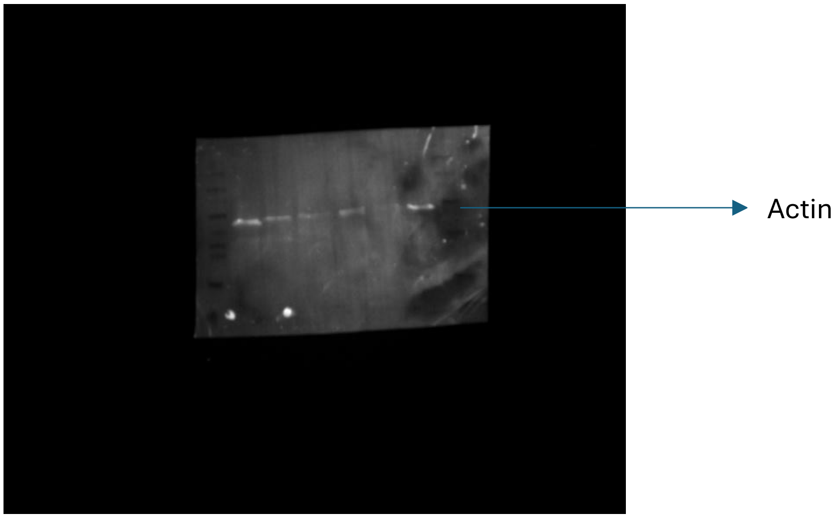

Technical replicate 3_caspase 3

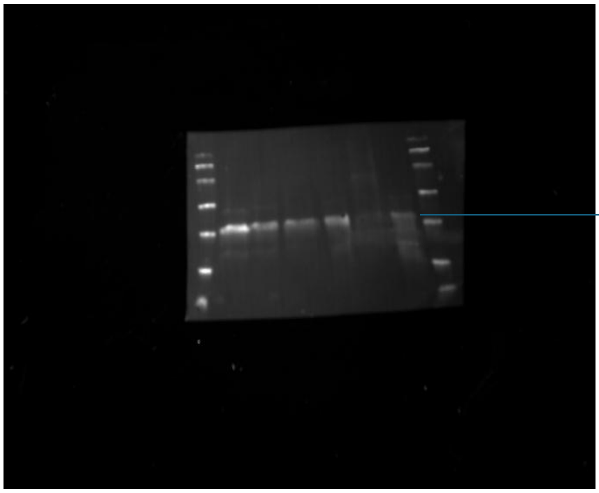

Caspase 3

Technical replicate 4_ actin

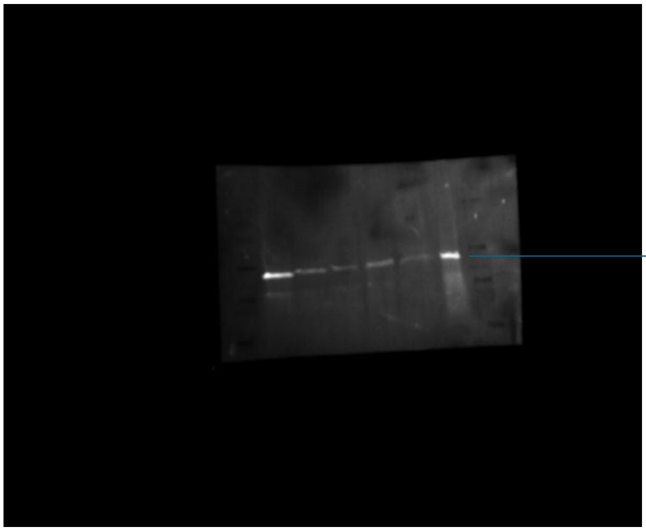

Actin

Technical replicate 4_caspase 3

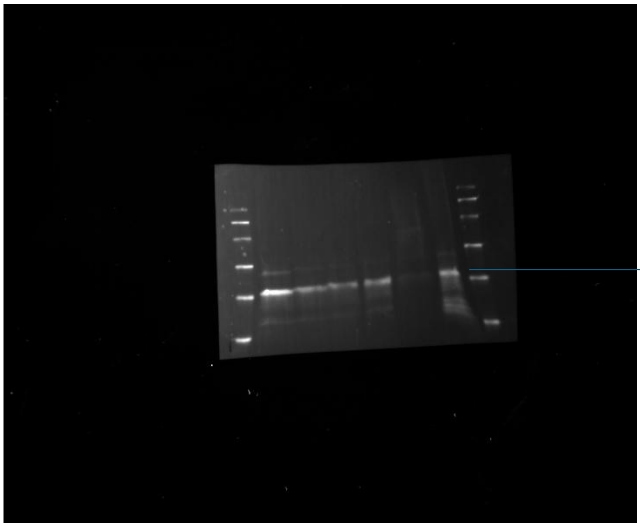

HT29 Apoptosis

Biological replicate 1_actin and caspase 3

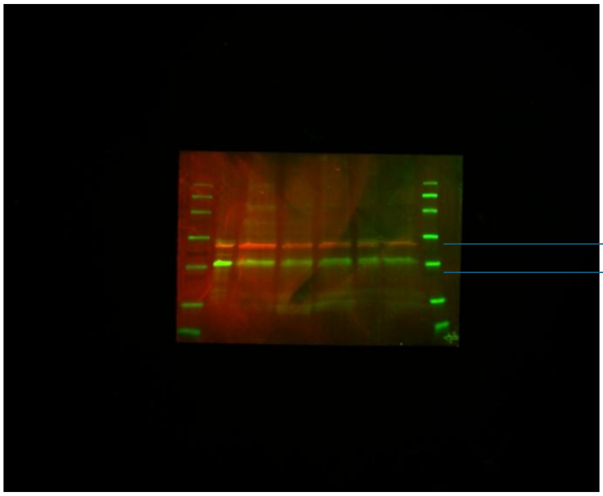

Biological replicate 2_actin and caspase 3

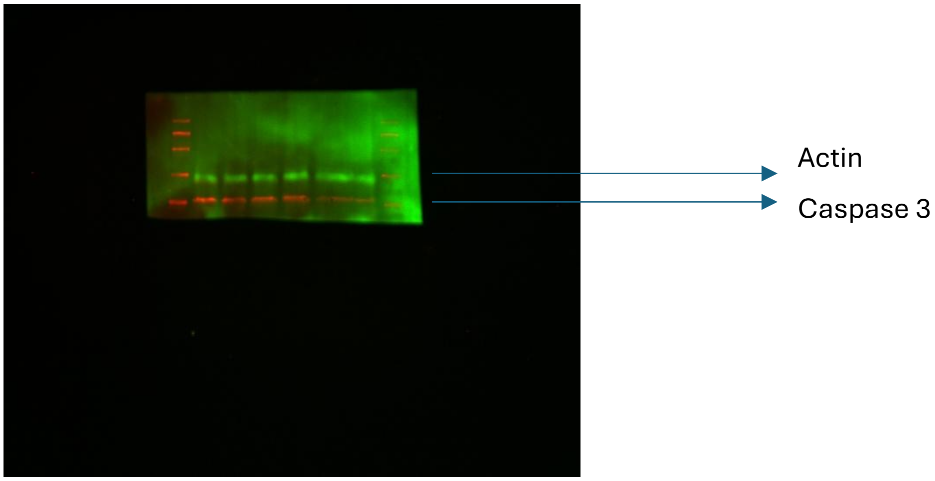

Biological replicate 3_actin

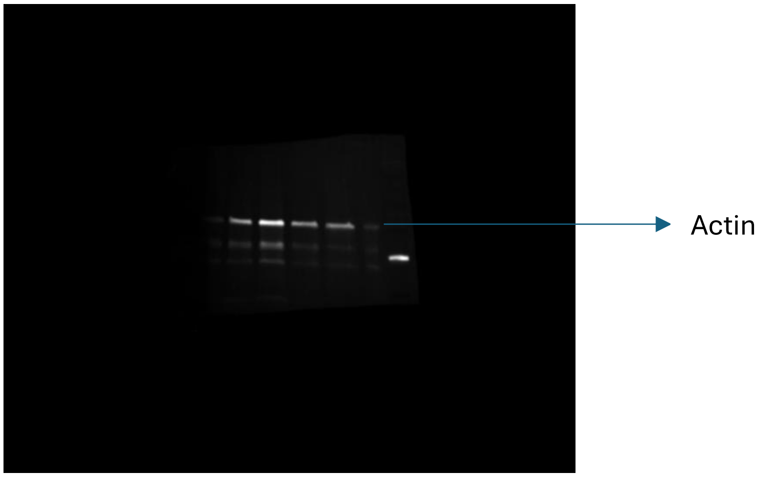

Biological replicate 3_procaspase 3

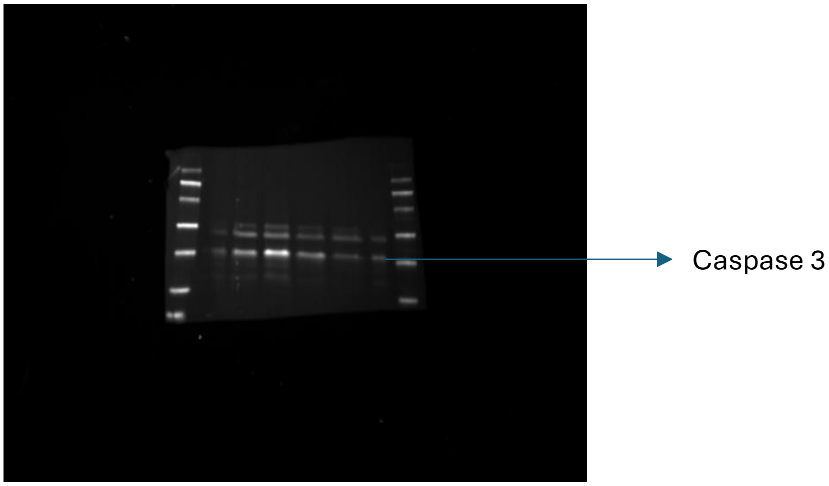

Technical replicate 1_actin

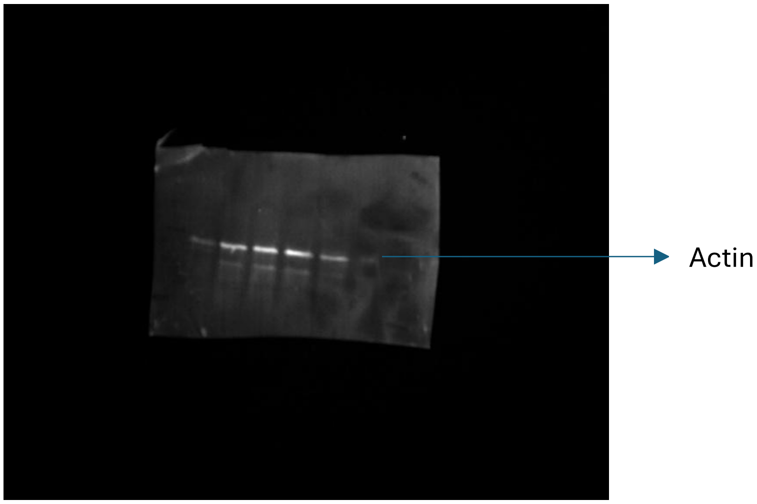

Technical replicate 1_caspase 3

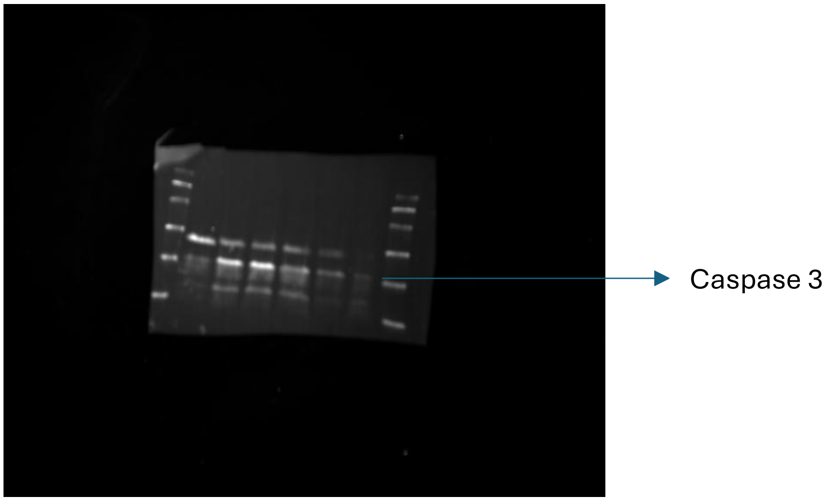

Technical replicate 2_actin

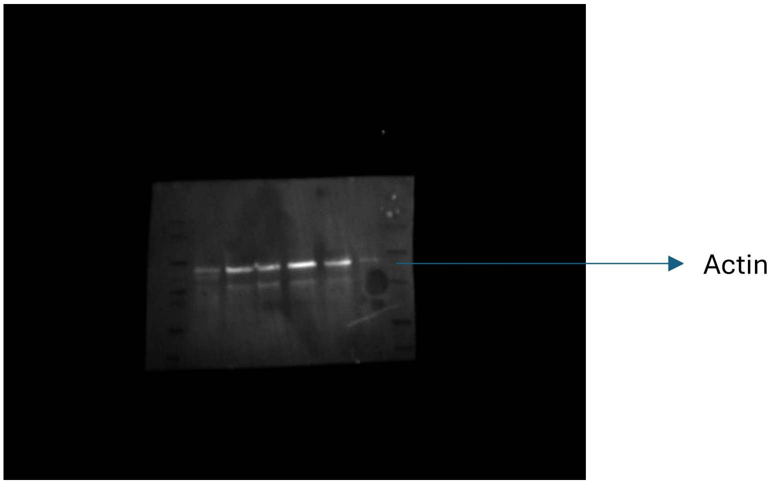

Technical replicate 2_caspase 3

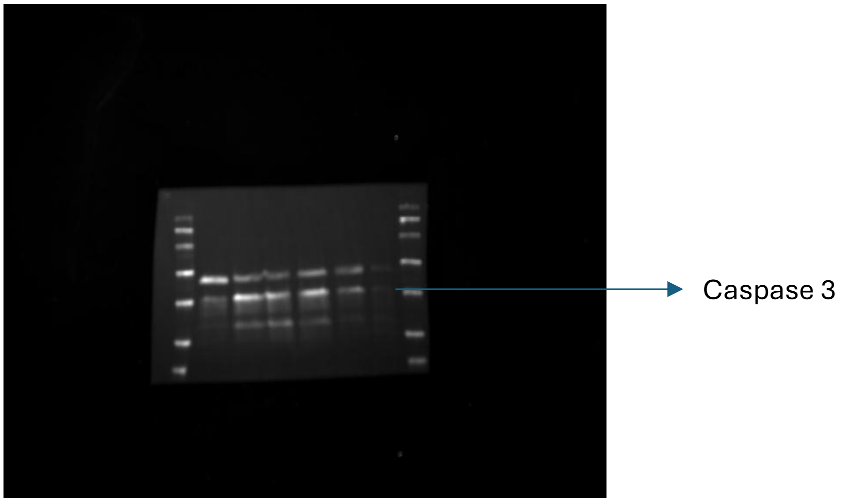

Technical replicate 3_ actin

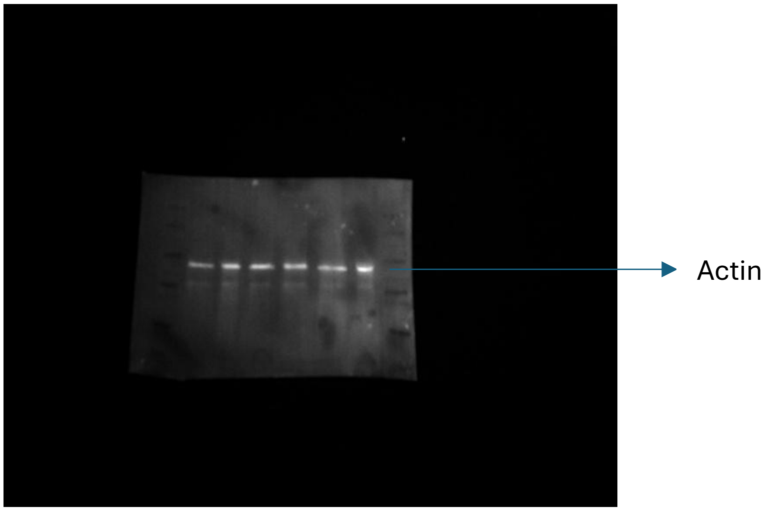

Technical replicate 3_caspase 3

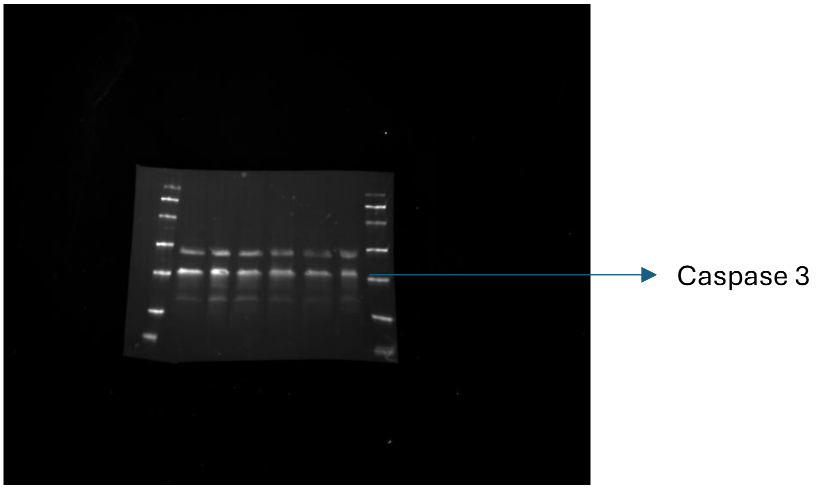

Technical replicate 4_actin

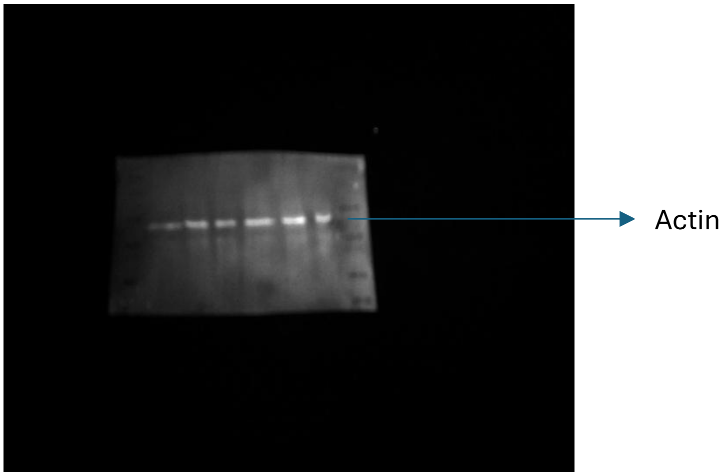

Technical replicate 4_caspase 3

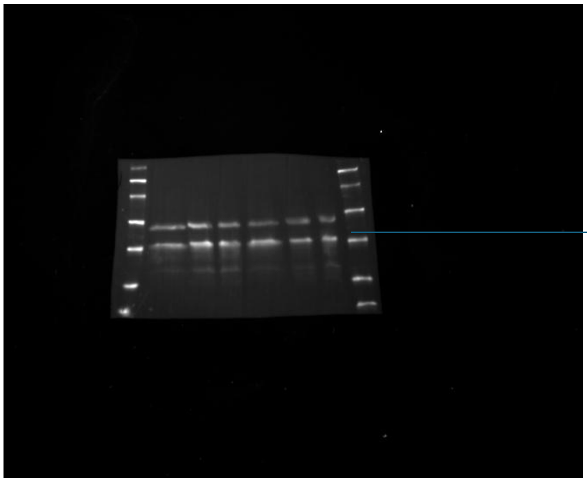

Caspase 3

HT29 Ferroptosis

Replicate 1_ ACSL4

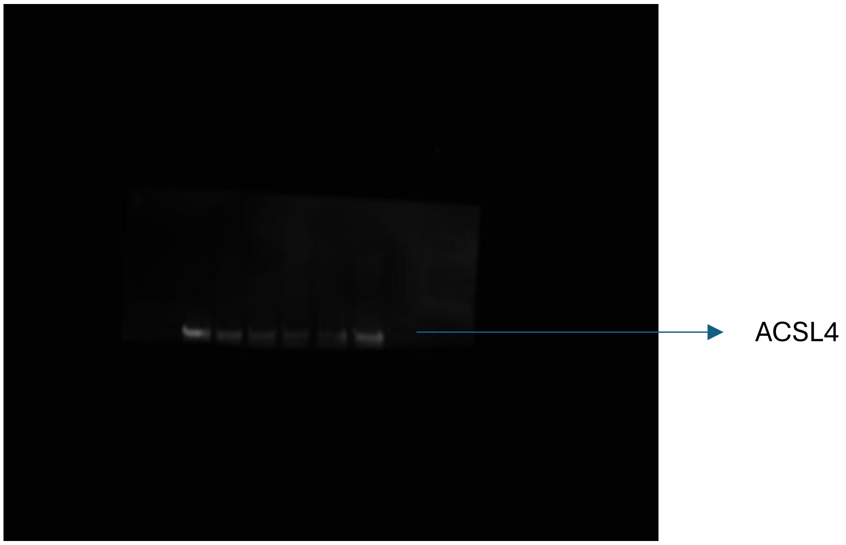

Replicate 1_ GPX4 and tubulin

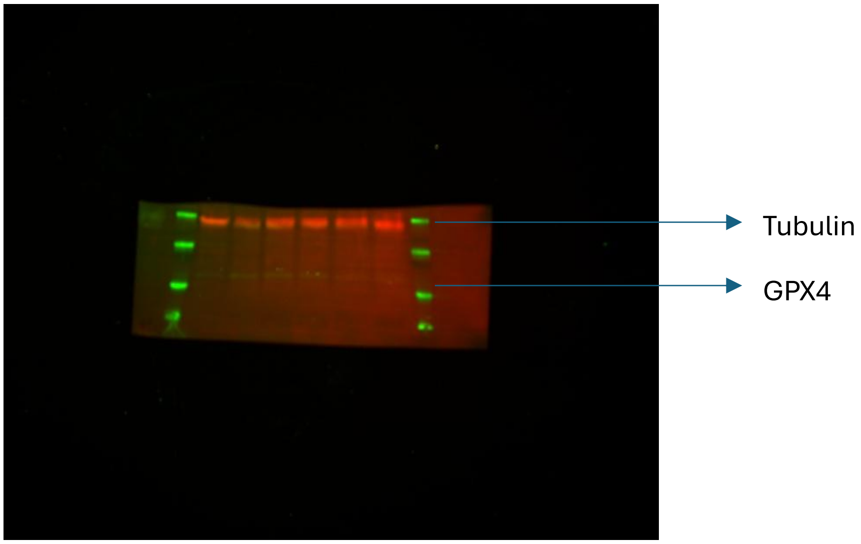

Replicate 2_ACSL4

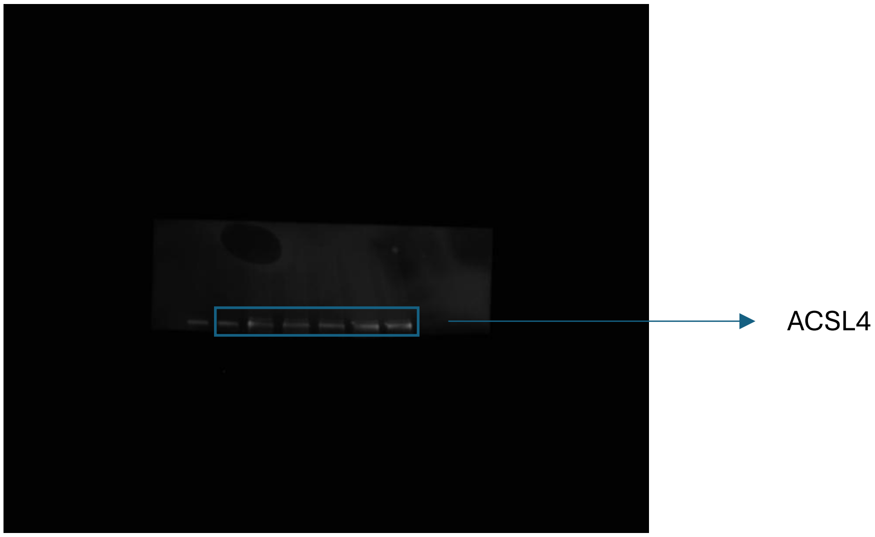

Replicate 2_GPX4 and tubulin

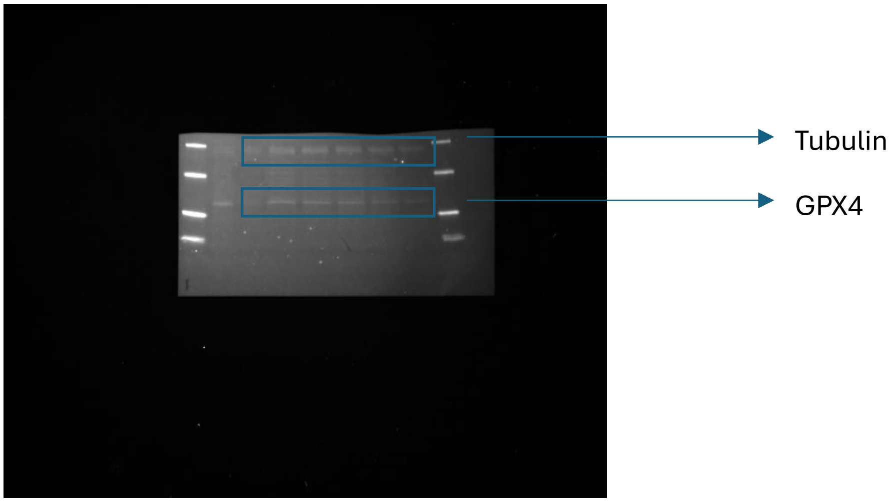

Replicate 3_ACSL4

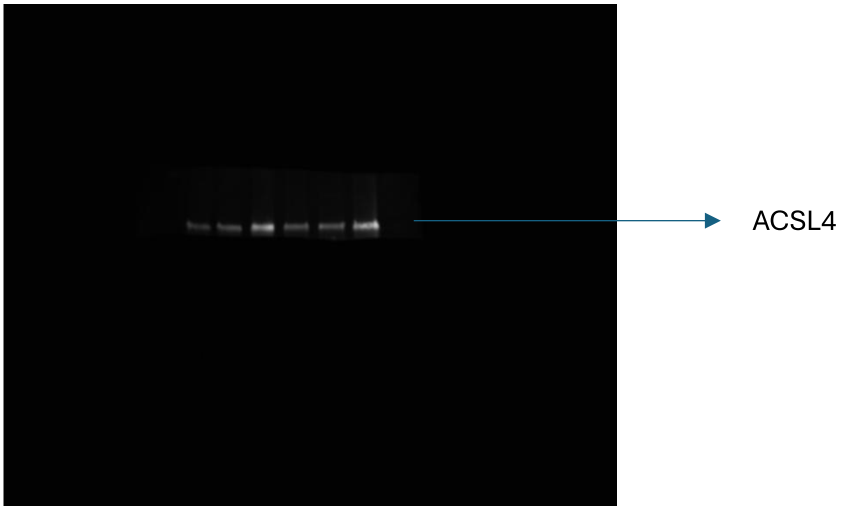

Replicate 3_GPX4 and Tubulin

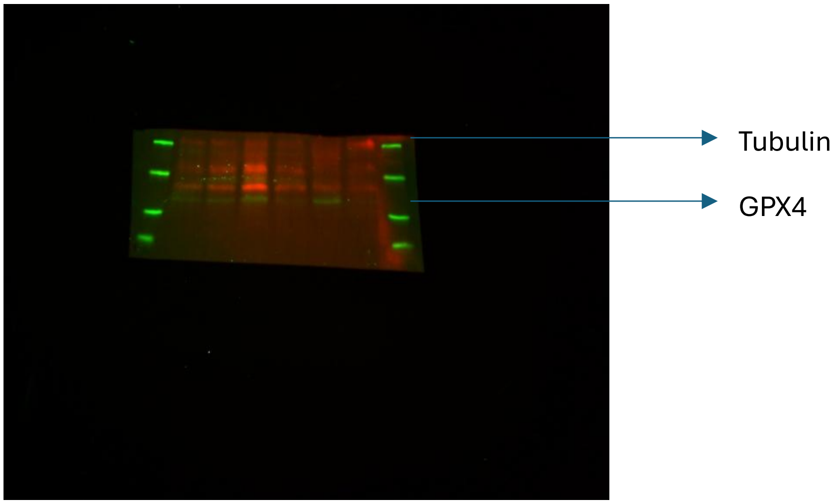

Supplement: Supplemental Material [file IPHB_A_2640678_SM0718.pdf]
